# Improved Inference of Taxonomic Richness from Environmental DNA

**DOI:** 10.1371/journal.pone.0071974

**Published:** 2013-08-26

**Authors:** Matthew J. Morgan, Anthony A. Chariton, Diana M. Hartley, Leon N. Court, Christopher M. Hardy

**Affiliations:** 1 Commonwealth Scientific and Industrial Research Organisation (CSIRO) Ecosystem Sciences, Canberra, Australian Capital Territory, Australia; 2 Commonwealth Scientific and Industrial Research Organisation (CSIRO) Land and Water, Kirrawee, New South Wales, Australia; University of Glasgow, United Kingdom

## Abstract

Accurate estimation of biological diversity in environmental DNA samples using high-throughput amplicon pyrosequencing must account for errors generated by PCR and sequencing. We describe a novel approach to distinguish the underlying sequence diversity in environmental DNA samples from errors that uses information on the abundance distribution of similar sequences across independent samples, as well as the frequency and diversity of sequences within individual samples. We have further refined this approach into a bioinformatics pipeline, Amplicon Pyrosequence Denoising Program (APDP) that is able to process raw sequence datasets into a set of validated sequences in formats compatible with commonly used downstream analyses packages. We demonstrate, by sequencing complex environmental samples and mock communities, that APDP is effective for removing errors from deeply sequenced datasets comprising biological and technical replicates, and can efficiently denoise single-sample datasets. APDP provides more conservative diversity estimates for complex datasets than other approaches; however, for some applications this may provide a more accurate and appropriate level of resolution, and result in greater confidence that returned sequences reflect the diversity of the underlying sample.

## Introduction

High-throughput pyrosequencing [1] has revolutionized the assessment of large-scale patterns of diversity by enabling easy access to large numbers of homologous long-read sequences amplified by PCR (“amplicons”) from complex environmental samples [2], [3], [4]. In addition to allowing researchers to deeply sequence individual communities, this technology permits simultaneous sequencing of many samples in parallel, making it suitable for complex experimental designs that incorporate replication to provide robust ecological inferences.

For many ecological applications of this technology, such as dietary analysis, food-web interactions, and biodiversity monitoring, the principal aim is to accurately infer the composition and diversity of communities in environmental samples [5], [6]. Roche 454 GS FLX Titanium pyrosequencing is the most widely-used technology for amplicon-based studies of eukaryote and prokaryote community diversity, but this approach generates three critical types of error: homopolymers, base substitutions and chimeras [7], [8], [9]. Errors generated from real sequences appear as novel diversity, and the failure to exclude them (“denoising”) leads to inflated diversity estimates in environmental samples [10], [11].

Current strategies for accommodating errors, including denoising software (e.g. AmpliconNoise [11] and Denoiser [12]), aim to maximize the information in the data; that is, maximize the proportion of the raw data that reflect the true diversity by removing errors completely or mapping them onto real sequences. While increasing the signal in a dataset is the primary reason for applying denoising algorithms, there remains an inherent trade-off between specificity (false positive rates) and sensitivity (false negative rates) as these approaches attempt to balance the removal of errors with the retention of real sequences. Distinguishing between real sequences and errors remains a significant challenge, particularly for low-abundance sequences, and can lead all denoising algorithms both to retain false diversity (False Positives) and incorrectly eliminate real diversity (False Negatives) [10], [11], [12], [13], [14], [15]. In particular, errors are known to inflate the diversity of low-abundance taxa [16] which complicates the study of “the rare biosphere” [17]. Unlike other scenarios such as genome assembly, deeper sequencing to improve sampling of rare taxa *increases* the number of observed errors and exacerbates the bias in diversity estimation [14]. Thus, accurately inferring the richness of an environmental sample is a significant challenge regardless of sequencing depth and underlying community diversity.

We have developed a new bioinformatic pipeline for removing errors from amplicon pyrosequences. The goal of all denoising programs is to retain real sequences regardless of rarity or abundance in order to obtain improved estimates of the true sequence diversity and community composition of environmental samples. Our approach uses the abundance distribution of similar sequences across independent samples, as well as the frequency and diversity of sequences within individual samples, to distinguish real sequences from errors. We have implemented our approach in Amplicon Pyrosequence Denoising Program (APDP) and tested it using four published 16S rRNA datasets previously used to benchmark amplicon denoising software [11], , and four low and high-diversity 18S rRNA datasets containing known sequences. We show that independently evaluating every observed sequence as valid or invalid, using prior knowledge about the nature and frequency of error-types, allows an increase in accuracy over existing strategies for both eukaryote and prokaryote communities. The output from APDP can be exported into existing software such as QIIME [19] and mothur [20] for downstream analysis of community diversity.

## Methods

### Overview of Error Removal with APDP

Amplicon Pyrosequence Denoising Program (APDP) is a Perl scripted pipeline which removes errors generated when PCR-amplified DNA fragments (amplicons) are sequenced using Roche GS FLX 454 Titanium pyrosequencing. The pipeline removes three critical error-types: i) DNA polymerase errors during PCR amplification and subsequently sequenced (causing base substitution errors); ii) chimeras formed during PCR amplification (creating a hybrid DNA molecule that registers as a novel sequence); and iii) pyrosequencing errors (generating base insertion or deletion errors, known as indels).

*Validation* of observed sequences is the central concept behind APDP – validated sequences are considered to be real sequences present in the original environmental DNA sample, and not generated by methodological errors. Observation of a sequence in a dataset, even in multiple independent replicates, is not sufficient to establish that it is a real sequence found in the environmental sample, as some sources of error such as high-frequency indel errors caused by pyrosequencing and chimera formation during PCR amplification [9] may be reproducibly generated in independent replicates if the same parent sequences are present. APDP uses the abundance distribution of similar sequences across independent samples (preferably technical or biological replicates), as well as the frequency and diversity of sequences within an individual sample, to distinguish valid observed sequences from errors observed in the dataset. Algorithms such as AmpliconNoise and Denoiser map (or cluster) reads onto inferred parent sequences and select a single sequence to represent all the reads in a cluster for downstream analyses [11], [12]. These approaches can infer incorrect sequences as parental. The consequences of this can be the retention of false diversity (e.g. [15]). We hypothesized that applying more conservative assumptions about the properties of errors will lead to improved parental sequence inference, and reduce the likelihood of retaining sequences that represent erroneous genotypes or OTUs.

APDP makes three assumptions about the properties of errors generated by Titanium amplicon pyrosequencing: Errors will i) co-occur with parent sequences among samples; ii) have fewer reads than parent sequences; and iii) have predictable read abundance relative to co-occurring parent sequences, according to error-type (substitution, indel, or chimera). APDP uses several parameters to describe the predicted abundance of different errors. The default values have been determined from multiple independent positive control data sets of eukaryote 18S rRNA gene amplicons, but are shown to also effectively remove errors for prokaryote 16S rRNA gene amplicons using this sequencing platform. All parameter values, however, can be defined by the user. The approach described here can be used with other high-throughput technologies, and appropriate parameters would need to be derived from control datasets from each platform. APDP has two versions: the default multi-sample version (APDP-MS) as described below, and a single-sample version (APDP-SS). APDP-SS uses a modified Preliminary Validation script ignoring the validation criterion requiring low-abundance sequences to appear in multiple samples.

### Description of the APDP Algorithm

APDP uses four discrete steps to fully denoise a dataset. Here we outline the steps, and a graphical representation of the workflow is presented in Fig. S1. Error containing reads are identified and removed at each step using the criteria described below. It should be noted that APDP has no single denoising step.

#### Step 1: Filtering bad reads and assignment of good reads to sequences and samples

The first step processes raw reads provided in FASTA file format. There is some debate as to how well quality scores reflect the accuracy of individual reads or sub-regions [12], [14], [21]. APDP therefore determines acceptable reads using different criteria: acceptable reads contain an exact match to two user-defined sequences, usually the forward and reverse primer (and multiplex identifier (MID) sequences if applicable). A read length cut-off can also be applied. Accepted reads are trimmed of primer and MID sequences, binned according to unique amplicon sequence and assigned to their sample of origin based on the MID(s).

The resulting set of unique sequences is then further filtered to remove sequences containing multiple primer sequences or ambiguous nucleotides (Ns), and sequences observed only once in the entire dataset (global singletons). Removing these prior to OTU clustering has previously been shown to improve accuracy of diversity estimates from low-diversity mock datasets where few real sequences will be represented by global singletons [15]. This criterion guarantees that APDP will miss real singleton sequences where they are present, and the impact of this is discussed later. Local singletons, i.e. sequences with multiple reads in the dataset but single observed reads in individual samples, are retained and may be validated by APDP. Thus, sequences can be represented by single reads in individual samples. The output is a table comprising a row for each unique sequence, its number of accepted reads in the entire data set, and the number of accepted reads in each sample (Fig. S1: Step 1). This contains the data used in subsequent validation steps.

#### Step 2. Preliminary validation by sequence distribution and abundance across samples

Filtered unique sequences are assigned to groups. Currently, APDP assigns sequences to groups based on the accession number of the best hit in GenBank as determined by the BLAST [22] or MEGABLAST [23] algorithm (the score of the best hit is recorded but not used by APDP: high-identity hits are not required). Sequences that do not return a hit are treated as unknown, non-target sequences and removed. However, APDP is fully compatible with other methods of group assignment, such as similarity clustering (e.g. [10]), to retain such sequences.

Sequences within each group are evaluated according to their distributions across different samples. It is expected that many erroneous sequences will be placed in the same group and be observed in the same samples as the parent sequences from which they are derived, but at much lower frequencies than the parent sequences (a user-definable parameter – see below). In contrast, multiple closely-related real sequences may be present in the same group, and it is expected that these will either occur in different samples or at higher frequencies than expected errors. The most abundant sequence in the group is provisionally accepted as valid unless i) it is only ever observed as a local singleton; or ii) it has fewer than 10 reads and is observed in only one sample. Thus to be validated, rare sequences (<10 reads in the dataset) must have at least three reads, be present in more than one sample, and have multiple reads in at least one sample. Each additional sequence in the group is retained if it is observed in different samples to the most abundant sequence, or co-occurs with the most abundant sequence at higher relative frequencies than probable errors of this sequence. The relative frequency is calculated for each sample as a user-defined proportion of the number of reads observed in that sample for the most abundant sequence. The cutoff frequency is conservatively set at 0.50. This ensures that high frequency sequences co-occurring with the most abundant sequence will still be validated, as will low-frequency sequences with different distribution patterns. APDP-SS uses a modified Preliminary Validation script that does not require low-abundance sequences to appear in multiple samples (thus only requiring two rather than three reads to be retained).

#### Step 3: Secondary validation by potential error-type and frequency within samples

Sequences are next evaluated within samples while ignoring groups. In this case, the expectation is that some errors generated from one or more real parent sequences observed in the same sample could be assigned initially into different groups. The three main sources of error (indel, substitution and chimeras) are expected to lead to specific types of sequence and occur at different frequencies [7], [8]. For each sample, sequences are ranked by relative abundance (sequences with a single read in the sample are considered ‘ambiguous’ or unknown), and all unique combinations of three-way alignments are performed using MUSCLE [24] in order of abundance: the two more abundant sequences are considered the parent sequences and a third less-abundant sequence a potential daughter sequence. The type and number of differences between all three sequences is noted, as well as the number of reads and compared against expectations of the frequency of these types of errors derived from positive controls to determine whether one or more sequences could be generated as an error of the others. If the two parent sequences differ only by indels, the less abundant sequence is considered a potential pyrosequencing error and flagged as invalid *in that sample*. It is not rejected at this stage, as it may be considered valid in other samples, and subsequently retained (see Step 4). If the two parent sequences differ by base substitutions, it is possible that one was generated as a DNA polymerase-error of the other. A sequence is flagged as invalid in that sample if it has fewer than *ax^n^* reads, where *a* is the number of reads of the more abundant sequence, *n* is the number of nucleotide mismatches inferred from the alignment, and *x* is a user-defined proportion (default value is 0.02). The potential for the daughter sequence arising as a chimera of the two parent sequences is assessed by comparing the match of the daughter sequence to the parent sequences moving from the start of the alignment towards the end. If the daughter mismatches both parents at any point, the probability of it being a chimera is considered to be zero. If that is not the case then the number of times that the daughter sequence ‘switches’ from matching one parent to the other is counted. The daughter is flagged as invalid if it has fewer than *by^s^* reads, where *b* is the number of reads of the less abundant parent, *s* is the number of “switches”, and *y* is a user-defined proportion (default value is 0.15). Sequences that pass all three tests are flagged as valid in the sample.

#### Step 4: Final validation

After all samples have been separately processed, the valid and invalid observations are collated for each sequence. The final step requires the user to define the number of valid observations required to retain a sequence. In cases where no technical replication has been performed (e.g. PCR or sequencing replicates), there is no justification to exclude any sequence validated in one or more samples. Sequences passing a threshold of valid observations (default = 1) are accepted.

### Datasets used to Benchmark APDP

We assessed the denoising accuracy of APDP using eight Roche 454 GS FLX Titanium datasets derived from eukaryote (18S) or prokaryote (16S) ribosomal RNA (rRNA) gene amplicons (Table S1). Six datasets comprised low-diversity constructed communities of known sequences (18Smock1-3, 18Smock4-6, 16Sv13, 16Sv34, 16Sv45 and 16Sv6). One (18Smock1-3) was used to develop APDP and set the default parameter values. Five datasets, including a replicate of the test set (18Smock4-6) and four 16S datasets, were used to test APDP under default parameters. To avoid the problem of over-training APDP to a single dataset, the additional artificial datasets were chosen from the literature to increase the variation in the gene target (18S rRNA vs 16S rRNA), 16S gene subregion (affecting level of variation between taxa, amplicon length, and expected error-profile), expected richness, taxonomic composition, sequencing depth, and laboratory of origin (Table S1). Datasets of known composition such as these are widely-used for benchmarking denoising approaches as it is possible to assess the accuracy both of the number of clusters returned and their identity [11]. However, such datasets are generally low-complexity and do not possess a long tail of rare taxa characteristic of environmental samples [11], [12], [14]. The remaining two datasets therefore comprised highly-diverse environmental communities of plankton and macrophytes sampled from along the Murray River, Australia (18SEnv1-2).

#### High-diversity datasets

Two complex environmental data sets were generated in this study from water column, sediment and vegetation samples taken at two sampling locations along the Murray River near Mildura, Victoria, Australia in austral Summer (7–9^th^ February 2009, 18SEnv1) and Autumn (24–26^th^ April 2009, 18SEnv2). At each location, sampling comprised: four 15 liter samples representing the water column (open water, littoral water, littoral near-bottom water, and aphotic near-bottom water), one sample of macrophytes and two (Autumn) or three (Summer) zooplankton net sweeps. No specific permissions were required by State and Federal authorities to collect water, macrophyte or zooplankton samples that were used in this study. This study did not involve endangered or protected species.Material from all samples (excluding zooplankton) was separated into size classes by passing through a series of filters (500–2000 µm, 53–500 µm, 1–53 µm, 0.22–1 µm). A total of 44 and 48 environmental samples were processed for 18SEnv1 and 18SEnv2 respectively. DNA was extracted from each sample using UltraClean Soil DNA kits (MO BIO Laboratories Inc, Carlsbad, CA). In addition, 18SEnv2 included positive control independently barcoded sample of total DNA extracted from a single freshwater shrimp to further assess the default settings. PCR reactions over 35 cycles to amplify between 200 and 500 base pairs (bp) in the 3′ region of the gene encoding the 18S ribosomal RNA were conducted on all samples using the primers All18SF (5′-TGGTGCATGGCCGTTCTTAGT-3′) and All18SR (5′-CATCTAAGGGCATCACAGACC-3′) as previously described [2]. PCR products were purified using Qiagen QIAquick PCR Purification Kit and amplified for a further four cycles to attach Titanium fusion primers incorporating sequencing adapters and a sample-specific combination of forward and reverse barcode sequences. A single PCR was performed for each sample from each site, labeled with a unique forward and reverse barcode combination. Tagged samples were pooled in equal concentrations (0.22–1 µm fractions were added at 1/5 concentrations due to small amplicon lengths that might preferentially sequence). Summer (18SEnv1) and Autumn (18SEnv2) samples were processed and sequenced separately on 1/2-picotiter plate each using Roche 454 GS FLX Titanium sequencing chemistry.

#### Low-diversity datasets

Two 18S rRNA amplicon datasets were created using pre-defined assemblages consisting of 16 cloned 18S rRNA gene sequences of varying length and distance, mixed in three different ratios (see Fig. S2 for experimental design and Table S2 for clone sequences). For each of the three different concentration assemblages (Fig. S2 Assemblages 1–3) we performed 5 separate PCRs (technical replicates) which were individually barcoded in triplicate following the procedure above. The resulting 45 samples were pooled into a single tube in equal concentrations. This pool was split into two and each half sequenced on ¼-picotitre plate using Roche 454 Titanium sequencing technology. This procedure generated two sequence replicates each containing three barcoded assemblages of 16 18S rRNA gene clones mixed in different concentrations (18Smock1-3 and 18Smock4-6).

Four 16S rRNA mock community datasets were selected to represent the diversity of regions used in prokaryote metagenomics (Table S1). The first 16S rRNA dataset (16Sv13) comprised twelve samples derived from pyrosequencing of the V1–V3 region from a mock community of 21 prokaryotes constructed to test the sequencing and analysis protocols used by the Human Microbiome Project [25] (Tables S1 and S3). Amplicon pools were sequenced in triplicate at each of four different sequencing centers, and twelve samples were chosen to incorporate three sequencing replicates performed at each of the four centers. Reference sequences were obtained by filtering the relevant region from the Human Microbiome Project Mock dataset alignment and trimming it to match the observed pyrosequence lengths for each denoising approach. We followed a previous study that used this dataset to benchmark denoising strategies and excluded *Methanobrevibacter smithii* sequences from the reference alignment [14]. These sequences show low similarity to the primers and were rarely amplified.

Two further 16S rRNA datasets (16Sv34 and 16Sv6) datasets comprised a set of V3–V4 and V6 region amplicons generated from an artificial community of 20 16S rRNA clones (V3V4P and V6P [18]). Reference sequences for both these datasets were obtained from the original authors (S. Craig Cary, personal communication).

The final Titanium pyrosequence dataset (16Sv45) was a published set of 16S rRNA V4–V5 region amplicons, generated by PCR of a mock community of 91 full length 16S rRNA clones in equal concentrations. This is currently the most diverse mock community used to benchmark Titanium denoising and diversity estimation tools. We used the modified set of 80 reference sequences used in [26] (Michael Rosen, personal communication). This set of references sequences accounts for a number of complications in the original reference sequences, including ambiguous base calls, indistinguishable sequences, and a high homopolymer rate for some sequences [26].

### APDP Analyses

All single-sample and multi-sample datasets were analysed using APDP-SS or APDP-MS respectively using default parameter values for Steps 1–3, with the following exceptions: 1) for the 16Sv13 dataset, many of the amplicons were too long to sequence through the reverse primer under the conditions of the original study. This resulted in many long reference sequences being rejected at the first filtering step and highlights a drawback of using APDP for analysing long amplicons where few reads will reach the reverse primer. Therefore for these datasets, we truncated pyrosequences at 200 bp and 400 bp prior to applying the regular filtering steps rather than search for a reverse primer; 2) the group assignment step for all datasets used MEGABLAST; 3) environmental sequences were not excluded for the 16S datasets, as this was found to slightly improve performance. The final validation step (Step 4) varied between multi-sample data sets. For the 18Smock data sets, sequences were retained if validated in four out of five technical replicates. For the 16Sv13 datasets, sequences were retained if validated in all three sequencing replicates from at least one of the four sequencing centers. For the 18SEnv data sets, there was no technical replication among the samples, so sequences were retained if validated in a single sample. Sequences in all single-sample datasets were retained if validated using default parameters.

### Benchmarking the Performance of APDP

The total number of sequences or OTUs returned is not a good criterion to evaluate the performance of an algorithm, as some real sequences may be falsely rejected and erroneous sequences falsely retained [27]. Therefore, we used both the accuracy of returned sequences and the accuracy of OTUs, to evaluate the performance of APDP.

Validated pyrosequences that perfectly matched a reference sequence were classified as true positives, following [26]. Terminal gaps were excluded from consideration, so that sequences that terminated early were counted as true positives. Pyrosequences within 3% pairwise distance (excluding ambiguous bases and terminal gaps) to a reference sequence with no closer match were classified as near-matches, similar to the criterion used in [18]. Previous studies have considered near-match sequences to be false-positives as they represent incorrectly called genotypes [26], whereas others have considered them close enough to be effectively true-positives [18] (for example, the clustering method used in [11] would cluster most, if not all, near-match sequences with the correct reference sequence). We differentiated between true-positive and near-match sequences in order to assess the affect of alternative measures of accuracy on the relative performance of APDP and other approaches. For each reference sequence only one pyrosequence could be considered a true-positive or near-match: if more than one pyrosequence hit the same reference, the closest match (or most abundant in the case of a tie) was considered a true-positive, and others classified as false positives. Reference sequences with no matching or near-matching pyrosequences were classified as false-negatives.

To construct OTUs, validated sequences were aligned in MUSCLE using default parameters followed by a refinement step. The dist.seqs command in mothur was used to make a pairwise distance matrix, and OTUs were constructed using the average-neighbor method. The most abundant pyrosequence was used as a representative for each OTU. OTUs were classified as true positives if the representative sequence matched a reference sequence. Similar to the classification scheme used [18], remaining OTUs were classified as miscalled if the representative sequence was within 3% of an otherwise missing reference and comprised pyrosequences matching a reference. OTUs were classified as near-match if the representative sequence was within 3% of a reference that otherwise would be missing and did not contain any pyrosequences matching a reference. As above, OTUs more than 3% different to a reference, or those where a closer match or more abundant OTU was assigned to the same reference were classified as false positives. References with no true-positive, miscalled, or near-match OTU, were classified as false negatives.

For the two 18SEnv data sets, although the composition of these datasets is unknown (apart from the positive control samples), observations during sampling and previous work demonstrated the presence of at most three freshwater shrimp decapod species in this section of the Murray River [28] (see Table S4). Numerous other taxa are present (e.g. diatoms, algae, fungi etc), but the actual diversity of these is unknown. The three decapod species represent the only detectable taxa for which the maximum number of species is known through independent observation. The 18S rRNA sequence for the amplicon used in this study is known for individuals of each species and they cluster into unique 3% OTUs. Although the genetic diversity within each species or individuals at this locus is not known, we consider it highly unlikely that each species will occupy more than one OTU because this region of the 18S rRNA gene is highly conserved between congeneric species: species in different genera often share identical sequences, and many genera are separated by <1% genetic distance. In this study, the 3% distance used to cluster sequences into OTUs is greater than the inter-specific genetic distance between each of the three shrimp species and at least one other species. Thus, 3% OTUs would be expected to encompass all intraspecific diversity. We therefore treated this subset of three predicted sequences (and three 3% OTUs) to be a surrogate for comparing the accuracy of denoising approaches for an entire complex dataset, and infer that poorly denoised decapod sequences would be reflected with the other taxa present that could not be reliably identified and evaluated in the same way. Validated sequences with a bitscore >200 to the best hit from a MEGABLAST search of the NCBI nr database were assigned the taxonomy of the best hit. The taxonomic identity and number of sequences assigned to decapod species was used to assess accuracy.

### Comparison with other Algorithms

We compared the performance of APDP with three widely-used denoising approaches. All six mock community datasets were analyzed with AmpliconNoise [11]. Raw flowgram files from 18Smock1-3, 18Smock4-6 and six 16Sv13 samples (representing three sequencing replicates from two sequencing centers) were processed separately with AmpliconNoise v1.27 using the protocol and parameter values in the example script provided with the software (RunTitanium.sh). The only deviations from this protocol were: 1) minflows was set to 120 for both 18Smock datasets; 2) the truncation length prior to running SeqNoise was set to 200 bp and 400 bp for 16Sv13, and to 130 bp for both 18Smock datasets; 3) we used Perseus rather than PerseusD for chimera checking; and 4) Perseus parameter settings of α = −6.14268 and β = 0.40297 [11] were used for the 16Sv45 chimera checking step. Denoised pyrosequences for the 16Sv34 and 16Sv6 datasets were obtained from the original authors (Charles Lee, personal communication). Denoised pyrosequences for 16Sv45 were obtained from http://userweb.eng.gla.ac.uk/christopher.quince/Data/Titanium_s25_cd.fa, and chimera-checked using Perseus as described above. Denoised pyrosequences from each dataset were then put through the final steps in the RunTitanium.sh protocol to find all unique sequences, construct 3% OTUs and return a set of representative sequences.

All eight data sets were analyzed with QIIME v1.3 (incorporating the Denoiser algorithm) and mothur v.1.22 (including the implementation of PyroNoise [29]; v.1.20 was used to error-correct both environmental data sets). For each analysis method, raw Titanium pyrosequencing data (as sff files) were analyzed using command pipelines and parameter settings based on available online recommendations from the relevant authors (QIIME tutorials homepage at http://qiime.org/tutorials/index.html and Schloss Lab Standard Operating Procedure [14] and available at http://www.mothur.org/wiki/Schloss_SOP). All datasets were denoised as independent sff files (for example 18Smock1-3, 18Smock4-6, 18SEnv1 and 18SEnv2 were analyzed as four separate files). The separate samples comprising 16Sv13 were denoised separately by each method, and then combined into a single file for further processing. Suggested parameter values were altered in some instances due to the nature of our datasets. For example, the suggested “minflows = 450” in the mothur SOP could not be used with the 18S rRNA datasets as our full-length amplicons are too short to meet this threshold. Other alterations were found to improve results over the suggested defaults, and the best performing set of parameters was used in our comparisons. The commands and parameters used for each analysis method and dataset are shown in Fig. S3 and Fig. S4.

The performance of each method was measured by evaluating the accuracy of individual denoised sequences and 3% OTUs using the same criteria as described above. OTUs were constructed from denoised pyrosequences a following the commands in Fig. S3 and Fig. S4. For the two high diversity 18SEnv data sets, the accuracy of each method was determined by the number and identity of decapod OTUs returned. A representative sequence was obtained for each OTU and taxonomy was assigned to each OTU as above.

## Results

### Performance of APDP

All data sets contained many more unique sequences than expected due to large numbers that were derived from errors (see number of unique sequences in Table 1). The observed relative frequency of known and reference sequences across all nine data sets ranged from 0.001% to 31.53%, and varied over several orders of magnitude within each data set (Tables 2, S4 and S5). Among data sets errors accounted for 16.73%–79.82% of accepted reads and 98.46%–99.86% of unique sequences. A significant number of relatively abundant errors were observed above the minimum frequency thresholds in all data sets, and most were correctly rejected (Table 2). Despite the high error rates, APDP was able to recover the vast majority of reference sequences correctly in all data sets. APDP performed particularly well on the multi-sample 18S rRNA datasets. The impressive performance of APDP here is unsurprising, as the 18S datasets closely resemble the dataset that the algorithm was trained on, and fulfill the replicated multiple-sample study design criterion that it is designed to exploit. APDP missed a total of twelve out of forty reference sequences in two single-sample datasets (16Sv34 and 16Sv6) and one out of twenty four in 16Sv13. We examined the frequency of the false negative reference sequences in the corresponding raw data, and found that seven were not present in the raw data and four were excluded as global singletons. A final false negative had six reads in a dataset with no replication (16Sv6) and was excluded in favor of a more abundant error that was classified as a false positive sequence.

**Table 1:** Accuracy of APDP for eight Titanium pyrosequence data sets.

	Expectedsequences	Pyrosequencing reads	Unique Sequences	Filtered sequences	Preliminary Validation	Final Validation
**High diversity**						
18SEnv1						
All	Unknown	357,432	99,303	18,559	1,164	929
Decapoda only	3			523	4	3
18SEnv2						
All	Unknown	314,414	52,048	13,684	990	841
Decapoda only	2			754	2	2
**Low diversity**						
18Smock1-3	16	268,874	26,675	6,088	55	16
18Smock4-6	16	275,876	24,934	5,874	63	16
16Sv13	24	268,818	12,793	4,144	1,127	34
16Sv34	20	75,447	9.135	1,056	57	17
16Sv45	80	62,873	13,831	1,321	112	93
16Sv6	20	53,653	1,040	266	56	19

Also shown are the number of raw pyrosequences, number of unique sequences, number of unique sequences after APDP filtering, and the number of unique sequences after Preliminary and Final validation.

**Table 2:** Accuracy and sensitivity of APDP to rare taxa in high and low-diversity communities.

Data Set	Minimum reads (data set)	Minimum reads (sample)	Minimum relative frequency	Reference sequence frequency	Expected sequences	Potentially valid sequences	FP	FN
18SEnv1	3	2	0.0008%	0.002%–2.465%	3	523	0	0
18SEnv2	3	2	0.0009%	0.015%–8.149%	2	754	0	0
18Smock1-3	8	2	0.0105%	0.04%–17.51%	16	1,381	0	0
18Smock4-5	8	2	0.0099%	0.05%–17.67%	16	1,364	0	0
16Sv13	6	2	0.0007%	0.13%–31.53%	24	1,276	11	1
16Sv34	2	2	0.0027%	0.001%–1.178%	20	1,344	4	7
16Sv45	2	2	0.0032%	0.021%–0.556%	80	1,330	13	0
16Sv6	2	2	0.0037%	0.002%–14.193%	20	311	3	4

For each data set the table shows the theoretical minimum number of reads required for a sequence to be potentially validated by APDP in the total data set and in each sample, the minimum relative frequency, the relative frequency of reference sequences, the expected number of sequences or OTUs, the number of unique sequences that passed the minimum read thresholds and were therefore potentially valid sequences, the number of errors retained (FP), and the number of undetected reference sequences (FN).

### Reproducibility of Sequences across Technical Replicates

The six assemblages in the two 18Smock datasets (18Smock1-6) were used to test the reproducibility of real and error sequences among technical (i.e. PCR) replicates. All 16 reference sequences were always observed in all five replicates. A number of errors (126–273 sequences) were observed in all five replicates, although most were present in just one or two replicates (Fig. S5). We examined the relationship between sequence abundance and technical reproducibility using all unique sequences observed in 18Smock6. Mean rank abundance was strongly correlated with the number of replicates in which a sequence was observed (*r* ^2^ = 0.86) and few sequences were observed in all technical replicates (Fig. S5). The rank-abundance of validated sequences also varied little across PCR replicates (Fig. S6). Importantly, the valid status of sequences was not dependent on rank abundance – for example one reference sequence ranked 113^th^ in read abundance in one technical replicate and was validated, whilst many more abundant errors were identified and removed (Fig. S6 C).

### Comparison with other Approaches

Over all six mock datasets, APDP returned the fewest false positive and false negative sequences (Table 3). APDP also returned the fewest false positive and false negative OTUs when only true positives were considered (“TP-only”). AmpliconNoise and mothur returned fewer false negative OTUs where miscalled and near-match OTUs were considered correct (“TP+NM”), and this came at the expense of higher false positive rate (Table 3). Excluding sequences and OTUs with fewer reads than the minimum detection threshold for APDP (“cutoff”; see Table 2 for threshold for each dataset) reduced the number of false positives (but still above APDP), and resulted in increased false negatives above APDP.

**Table 3:** Overall accuracy of denoised pyrosequences and 3% OTUs retained by APDP and three alternative denoising approaches using six artificial community datasets.

				Sequences				3% OTUs			
				TP only		TP+NM		TP only		TP+MC+NM	
Method	Number of Datasets	Total Expected Sequences	Total Observed Sequences	FP	FN	FP	FN	FP	FN	FP	FN
APDP	6	176	195	**43**	**24**	**31**	**12**	**19**	**21**	**9**	11
AmpliconNoise	6	174	506	383	50	360	27	189	39	164	14
cutoff	6	174	172	63	65	48	50	59	46	44	31
mother	6	183	6205	6052	38	6038	24	2358	35	2329	**6**
cutoff	6	183	538	385	39	377	31	315	36	292	13
QIIME	6	204	1113	1024	115	946	37	431	102	351	22
cutoff	6	204	772	683	115	610	42	269	102	190	23

Bold numbers indicate best result in the column. The number of false positives (FP) and false negatives (FN) is shown for cases where miscalled and near-match OTUs are considered to be incorrect (TP only) or correct (TP+NM and TP+MC+NM). In addition, the results are shown for each method after excluding OTUs with fewer reads than the minimum detection threshold of APDP (cutoff).

For denoised sequences returned from individual datasets, APDP returned generally low false positive and false negatives for all multi-sample and most single-sample datasets (Tables 4 and 5). When only true positive sequences were considered correct (“TP Only”) APDP had the fewest false positives in five of six datasets, the fewest false negatives in four of six datasets, and the fewest for both in three of six datasets. APDP performed best by both methods in all multi-sample datasets. When true positive and near-match sequences were considered correct (“TP+NM”), APDP had the fewest false positives in four of six datasets, the fewest false negatives in four of six datasets, and the fewest for both in all multi-sample datasets. AmpliconNoise was the best alternative method, with fewer false negatives than APDP in two datasets (16Sv34 and 16Sv6) but more false positives in all datasets. Applying a read abundance cutoff to the alternative methods reduced the false positive rate considerably, but often increased the false negative rate above that of APDP (Tables 4 and 5). The only method to outperform APDP by both measures for a single dataset was AmpliconNoise after removal of singletons from the 16Sv34 dataset, but there was no single approach that consistently outperformed APDP by either measure. Removing low-abundance sequences from the AmpliconNoise results returned fewer false positives than APDP in some cases.

**Table 4:** Accuracy of denoised pyrosequences from the three multi-sample artificial community datasets retained by APDP and three alternative denoising approaches.

	18S1–3				18S4–6				16Sv13			
	TP only		TP+NM		TP only		TP+NM		TP only		TP+NM	
Method	FP	FN	FP	FN	FP	FN	FP	FN	FP	FN	FP	FN
APDP	**0**	**0**	**0**	**0**	**0**	**0**	**0**	**0**	**11**	**1**	**11**	**1**
AmpliconNoise	33	6	30	3	38	3	37	2	243	3	243	3
cutoff	2	12	2	12	0	10	0	10	36	3	36	3
mothur	2226	0	2226	0	2502	0	2502	0	742	9	738	5
cutoff	110	0	110	0	102	0	102	0	20	9	19	8
QIIME	188	2	186	0	172	1	171	0	496	34	476	14
cutoff	87	2	85	0	91	1	90	0	357	34	341	18

The number of false positives (FP) and false negatives (FN) is shown for near-match sequences are considered to be incorrect (TP only) or correct (TP+NM) sequences. In addition, the results are shown for each method after excluding denoised sequences with fewer reads than the minimum detection threshold of APDP (cutoff).

**Table 5:** Accuracy of denoised pyrosequences from the three single-sample artificial community datasets retained by APDP and three alternative denoising approaches.

	16Sv34				16Sv45				16Sv6			
	TP only		TP+NM		TP only		TP+NM		TP only		TP+NM	
Method	FP	FN	FP	FN	FP	FN	FP	FN	FP	FN	FP	FN
APDP	**10**	13	4	7	17	**4**	13	**0**	**5**	6	**3**	4
AmpliconNoise	20	16	9	**5**	28	17	22	11	21	**5**	19	**3**
cutoff	12	16	**2**	6	**5**	17	**1**	13	8	7	7	6
mothur	51	**11**	51	11	448	10	438	0	83	8	83	8
cutoff	20	**11**	20	11	99	10	92	3	34	9	34	9
QIIME	19	19	11	11	105	52	59	6	44	7	43	6
cutoff	19	19	11	11	86	52	41	7	43	7	42	6

Nomenclature and abbreviations are as Table 4.

The results were similar for 3% OTUs constructed from denoised pyrosequences from each approach (Tables 6 and 7). When only true positive OTUs were considered correct (“TP Only”) APDP had the fewest false positives in all six of six datasets, the fewest false negatives in five of six datasets, and the fewest of both in all multi-sample and two single-sample datasets. When true positive, miscalled, and near-match OTUs were considered correct (“TP+NM”), APDP had the fewest false positives in five of six datasets, the fewest false negatives in four of six datasets, and the fewest of both in all multi-sample and one single-sample dataset. As for the denoised pyrosequences, AmpliconNoise was the best alternative method, with fewer false negatives than APDP in two datasets (16Sv34 and 16Sv6). Again, applying an abundance cutoff to the alternative methods reduced the number of false positives, but increased the number of false negatives. However, false positives were reduced below the APDP level in only one dataset, and only when miscalled and near-match OTUs were considered correct (16Sv34 TP+NM).

**Table 6:** Accuracy of 3% OTUs constructed from denoised pyrosequences from the three multi-sample artificial community datasets retained by APDP and three alternative denoising approaches.

	18S1–3				18S4–6				16Sv13			
	TP only		TP+NM		TP only		TP+NM		TP only		TP+NM	
Method	FP	FN	FP	FN	FP	FN	FP	FN	FP	FN	FP	FN
APDP	**0**	**0**	**0**	**0**	**0**	**0**	**0**	**0**	**2**	**0**	**2**	**0**
AmpliconNoise	14	7	9	2	17	7	13	3	98	1	97	0
cutoff	2	10	2	10	2	9	1	8	30	1	29	0
mother	1017	1	1016	0	1021	0	1021	0	71	1	70	0
cutoff	97	1	96	0	101	0	101	0	4	1	3	0
QIIME	73	4	69	0	70	1	69	0	144	13	131	0
cutoff	37	4	33	0	36	1	35	0	82	13	69	0

The number of false positives (FP) and false negatives (FN) is shown for cases where miscalled and near-match OTUs are considered to be incorrect (TP only) or correct (TP+NM). In addition, the results are shown for each method after excluding OTUs with fewer reads than the minimum detection threshold of APDP (cutoff).

**Table 7:** Accuracy of 3% OTUs constructed from denoised pyrosequences from the three single-sample artificial community datasets retained by APDP and three alternative denoising approaches.

	16Sv34				16Sv45				16Sv6			
	TP only		TP+NM		TP only		TP+NM		TP only		TP+NM	
Method	FP	FN	FP	FN	FP	FN	FP	FN	FP	FN	FP	FN
APDP	**8**	**10**	3	5	**4**	**6**	**1**	**3**	**5**	5	**3**	3
AmpliconNoise	19	13	9	3	22	7	19	4	19	**4**	17	**2**
cutoff	11	13	**2**	4	5	7	2	4	9	6	8	5
mothur	20	10	12	**2**	138	17	124	3	91	6	86	1
cutoff	10	10	3	3	60	15	49	6	43	7	40	4
QIIME	16	15	4	3	77	40	41	4	23	12	19	8
cutoff	16	15	4	3	63	40	27	4	22	12	18	8

Nomenclature and abbreviations are as Table 6.

APDP was least effective on the 16Sv34 dataset (Tables 5 and 7), however all methods tested had difficulty identifying rare reference sequences (Table S6). This dataset returned fewer accepted reads than other datasets used in our study, possibly due to the expected length of the amplicons combined with a poor sequencing run (Table S1). We explored the hypothesis that APDP performs poorly on longer amplicon sequences by comparing APDP and AmpliconNoise on the same set of six 16Sv13 samples truncated at 200 bp and 400 bp (Table S7). APDP returned fewer false positive sequences and OTUs and fewer false negative sequences than AmpliconNoise at both truncation lengths. Both methods found at least a near-match to all expected OTUs.

**Figure 1:**
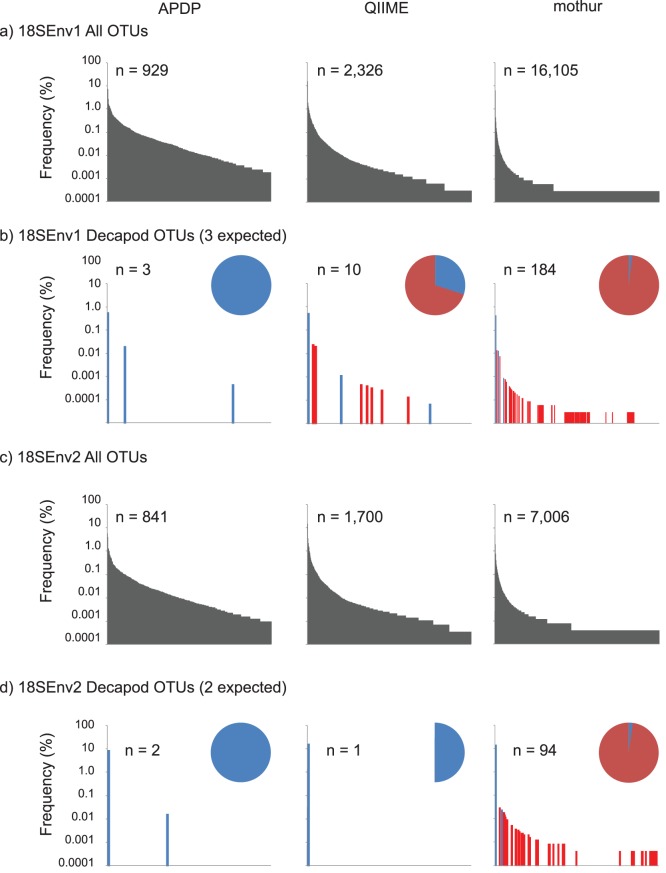
Abundance and accuracy of OTUs recovered by APDP, QIIME and mothur analyses of high diversity environmental datasets. Sequences are ordered by rank abundance on the x-axes. Note that the scale varies for each graph as the number of OTUs recovered differs for each dataset and method. Y-axes are log-scaled. The total number of OTUs recovered (n), and the relative frequency distribution for all OTUs (rank abundance bar chart) are shown for each analysis. a) All OTUs recovered from the high-diversity 18SEnv1 dataset that contains an unknown number of OTUs by each analysis method. b) OTUs recovered from 18SEnv1 that were classified as Decapoda (expected to comprise three OTUs). Spacing between some high-frequency bars has been manipulated to aid visualization of the mothur result due to the high OTU return. c) All OTUs recovered from the high-diversity 18SEnv2 dataset. d) OTUs recovered from 18SEnv2 that were classified as Decapoda (expected to comprise two OTUs). The expected number of decapod OTUs (b and d) is shown in parentheses along with a pie chart showing the proportion classified as good (blue), noise (red) or missed (no color).

In the high-diversity environmental samples, APDP recovered far fewer OTUs than the strategies implemented in QIIME or mothur and all three methods retained tails of rare OTUs (Fig. 1 A and C). To determine the relative accuracy of the diversity estimates in our two complex environmental datasets where the true compositions were unknown, we examined the subset of all OTUs recovered by each method that were classified as Class Decapoda (see Methods for justification of this approach). APDP recovered the correct number and identity of known true decapod sequences from within a diverse mixture of sequences in both 18SEnv1 and 18SEnv2 (Table S8), even when present in the raw data at low-frequency (Table S3). In contrast, mothur inferred 184 and 94 decapod OTUs for 18SEnv1 and 2 respectively, while QIIME inferred ten decapod OTUs in 18SEnv1 and one only from 18SEnv2. Both methods correctly recovered at least one OTU representative for each of the known species, except QIIME failed to recover an OTU representative of the shrimp *Paratya australiensis* from 18SEnv2 (Table S8) even though the sequence was present in four samples at relative frequencies of 0.002%–0.08% (Table S3). The majority of the false diversity recovered by each method was low-frequency OTUs (Fig. 1) that were inferred to be derived from errors of the true shrimp sequence present in the samples (Table S8).

## Discussion

Errors in high-throughput amplicon pyrosequencing of environmental samples can lead to inflated taxonomic richness estimates and distort inferred community composition which restricts the utility of new sequencing technologies when addressing ecological questions. Here, we present new software (APDP) for identifying real sequences and removing errors from Roche 454 FLX Titanium amplicon pyrosequences. The aim of APDP is to identify an error-free set of sequences that can be used alone or in conjunction with other software, such as QIIME and mothur, to test ecological hypotheses regarding the composition of eukaryote and prokaryote communities by significantly reducing or eliminating the contribution of erroneous diversity. APDP does not assign taxonomy to sequences, nor does it cluster sequences to create OTUs, but the resulting set of validated sequences can be used for this purpose to account for intraspecific variation rather than methodological error.

### Accuracy of Error-removal

The results presented here highlight the strengths and potential issues that arise from the conservative approach of APDP. In particular, the performance of APDP on a wide range of datasets using default error-frequency parameter settings shows the broad utility of this approach to remove errors and identify real diversity in prokaryote and eukaryote Titanium amplicon pyrosequencing datasets (Tables 3, 4, 5, 6 and 7). In addition to relatively low false positive and false negative rates, APDP has several other beneficial features: 1) it can process raw reads containing forward and reverse primers and multiplex identifiers; 2) is able to process >2M Titanium reads (i.e. all the reads from two full sequencing runs) distributed across >200 samples on a standard desktop computer with a single processor; 3) implements an error removal strategy that is not specific to any DNA target amplicon; and 4) does not require a reference alignment.

### Sensitivity and Specificity of APDP

A potential issue of the stringent error-removal strategy in APDP is the sensitivity to rare diversity. The ability to detect real rare sequences and distinguish them from low abundance errors is essential for accurate diversity estimation. The relative frequency threshold of detection by APDP is dependent on experimental design, but the minimum absolute frequency is fixed at two (single sample datasets) or three reads (Table 2). Importantly, this absolute lower bound is independent of the total number of reads, so increasing sequencing depth means increasingly rare sequences can be validated by APDP. Using these thresholds, APDP identified reference sequences within or below published detection limits for rare taxa (0.01%–1.0% [30], [31]) in both high and low-diversity data sets (lowest relative frequency = 0.0008%). Furthermore, our results show that existing widely-used strategies for error-removal often retain false low-abundance sequences and OTUs in high and low diversity datasets that can compromise inferences of the richness and extent of the rare biosphere (Fig. 1, Tables 4, 5, 6 and 7).

Although APDP performs well here on a range of datasets, there may be cases, such as under-sampled high-diversity heterogeneous communities, where real rare sequences will be missed due to the relatively conservative nature of the algorithm. The results presented here suggest that APDP will have greater specificity than other methods (it retains less false positives in most datasets), but this potentially traded for reduced sensitivity (it falsely rejects more true positives in some datasets). In particular, sequences observed only once in an entire dataset (i.e. global singletons) were missed by APDP analyses of 16Sv34 and 16Sv6. Singleton sequences comprise a large proportion of all metagenomic datasets, independent of the level of diversity in the underlying sample, and a large proportion of these are likely to be errors (Table 1). However, APDP will also remove real singletons where they are present. Removing singletons is a conservative approach and is based on our view that independent reproducible observation offers a greater degree of confidence that an observation is correct. This position is supported by our results (Fig. 1, Tables 4, 5, 6 and 7) and previously published studies (e.g. [15]) that all methods will have issues with effectively distinguishing real rare diversity from errors. When choosing an analysis method there is a trade-off between detecting real low-abundance sequences and rejecting low-abundance errors (Tables 4, 5, 6 and 7), and preference for either will depend on the biological question being addressed. This choice will be influenced by whether false positives or false negatives more strongly affect the biological conclusions drawn. If investigators are interested in rare sequences that may have been falsely excluded, APDP makes these sequences available as supplementary files for further investigation. Furthermore, missed rare taxa can be validated by sequencing more deeply whereas false identified taxa leave no obvious indicator that they are incorrect; importantly, our results suggest that APDP is less susceptible to the OTU inflation that affects other tools when deeply sampling low-diversity datasets [14].

### Repeatability of Real Sequences in Biological and Technical Replicates

APDP performs particularly well when biological and technical replicates are included in the experimental design (Tables 4 and 6). The use of repeated observation of sequences or OTUs across samples is not in itself novel, and the occurrence of OTUs across multiple biological replicates has been used previously to improve confidence in diversity estimates [32]. APDP has been designed to take advantage of both biological and technical replicates (e.g. independent PCRs from the same sample), and we strongly advocate the use of technical replicates to improve the identification and removal of errors. The majority of errors are rare and poorly reproducible between technical replicates due to the stochastic nature of the processes that govern their generation, whereas real sequences and more common errors are more abundant and highly reproducible (Fig. S5). APDP exploits this to remove errors: highly-abundant, reproducible errors are removed from individual replicates as their high read counts accurately fit assumed error profiles. Low-abundance, less reproducible errors, such as highly-divergent chimeras and multi-base DNA polymerase-errors, are more difficult to identify as errors in individual replicates but are removed by requiring sequences to be validated in a minimum number of replicates. When assessing the diversity of low-abundance sequences in high-diversity samples (i.e. sequences present with fewer than 10 reads across an entire experiment), APDP is dependent on the observation of rare real sequences in environmental or technical replicates. Low-depth sequencing of highly-diverse communities may impact on the ability of APDP to adequately define the composition of the rare biosphere, particularly when many of these taxa will be present at frequencies below 1/R, where R is the number of reads from each technical replicate. Here, real rare sequences may be observed in single samples at very low frequencies due to stochastic sampling effects, and these types of sequences can be excluded by APDP. However, evidence from incomplete sequencing of highly-diverse environmental samples has shown that real rare sequences (<0.020%–0.025% relative frequency per sample) are reproducibly observed in pyrosequencing datasets in both biological and technical (PCR) replicates [33]. In addition, our results confirm that real rare sequences are highly reproducible among technical replicates at similar relative frequencies (≥0.04%, Table S5). While technical replication is clearly desirable, it is not always essential for accurate error-removal using APDP: the 18SEnv1–2 environmental data sets have multiple biological samples but no technical replication, and three of the 16S datasets comprise a single sample with no replication, yet APDP was able to remove almost all errors and retain most of the correct sequences and OTUs (Fig. 1, Table 2). This supports our conclusion that APDP, combined ideally with appropriate experimental design incorporating multiple samples per treatment and replicate PCRs, is an effective approach to removing erroneous diversity and retaining real rare sequences in low and high diversity environmental datasets.

### Potential Sources of Bias and Inaccuracy

The current version of APDP requires that the entire amplicon (including reverse primer and/or MID) be sequenced. In the case of long amplicons (e.g. 16Sv45 data set amplicons are >350–400 bp), premature sequence termination and higher error rates [8] may lead to the exclusion of a high proportion of raw pyrosequences, although APDP is still able to remove errors from these data sets as effectively as the other tested algorithm (Fig. 1, Table 3). However, in the case of amplicons that are so long the reverse primer is unlikely to be sequenced (e.g. some 16Sv13 amplicons are over 500 bp), APDP failed to recover any reference sequences longer than 480 bp (data not shown). Truncating these sequences using a modified initial filtering approach, we were still able to retain more real diversity and had the same or better false negative rate than AmpliconNoise at both truncation lengths (Table S7). In addition, both methods performed better when amplicons were shorter, possibly because the first 200 bp of a pyrosequence are the most accurate, or because more 16S gene copies were indistinguishable from each other. In either case, it appears that preference should be given to identifying the shortest amplicon that provides adequate resolution to address the biological question at hand. Refining this approach to enable APDP to process partial sequences from long amplicons where the entire molecule cannot be sequenced with the current technology is possible, although improvements in read length are expected to significantly reduce the need for this feature.

A further possible drawback to our initial approach is the use of the BLAST algorithm in APDP to assign sequences to groups. By using this algorithm, APDP is in effect a semi-supervised approach, and the results of APDP could potentially be less accurate if applied to communities from novel environments with few close relatives present in the reference database. In addition, BLAST is relatively slow and takes up a large proportion of the total processing time (Table S9). We therefore re-analyzed four mock community datasets (18Smock1-3, 18Smock4-6, 16Sv13, and 16Sv45) using the 2% single-linkage precluster method (SLP [10]) to assign sequences to groups (Table S10). Using this unsupervised clustering approach, APDP again recovered the sixteen correct sequences and removed all errors in both 18S datasets. For the 16S datasets, fewer sequences were returned using SLP, which resulted in a loss of sensitivity to individual sequences and fewer false positives. This approach also recovered the same expected OTUs additional false positive OTUs (Table S10). This result was still better than any of the other methods tested using this dataset, including AmpliconNoise (Tables 4, 5, 6 and 7). APDP can, therefore, be used independently of a reference database to accurately retain real diversity and remove more erroneous sequences than all other tested approaches on these eukaryote (18S rRNA) and prokaryote (16S rRNA) datasets using the default error frequency parameter values.

### Implications for Ecological Applications

There are clear implications for ensuring accurate denoising in ecological applications where the accurate identification of organisms (or genotypes) present in environmental samples is of paramount importance, such as monitoring specific taxonomic groups. In such cases, this emerging sequencing technology will only be useful if the correct taxonomic diversity can be inferred from environmental samples. For example, environmental health of rivers is often assessed by conducting surveys of invertebrate diversity and accurate inference of taxonomic richness is likely to benefit from emerging molecular techniques [34]. Similarly, there are increasing efforts to describe the diversity present in different environments [35], in order to gain critical insights into ecological and biological processes affecting the distribution of organisms and communities within ecosystems [36]. Furthermore, accurate inference of genetic diversity is increasingly important in defining conservation units [37]. In our study, APDP outperformed the existing denoising approaches when inferring the known diversity of prokaryote and eukaryote datasets. Therefore it is an appropriate tool for estimating the taxonomic richness of environmental samples, and subsequently, for making inferences about the health and biology of environmental systems founded on robust ecological data.

## Conclusions

Accurate diversity estimation from Roche 454 GS FLX Titanium amplicon pyrosequencing requires that errors generated by the methodological pipeline be distinguished from true sequence diversity. APDP is an alternative denoising approach that can explicitly use the experimental design of a sequencing project to more accurately predict the number and identity of abundant and rare real unique sequences in multi-sample mixtures of known composition as well as complex environmental samples. APDP is written in Perl and runs on Linux using a command-line interface. Although used here on ribosomal RNA sequences, and Roche GS-FLX 454 Titanium chemistry, the approach used in APDP is applicable to any gene marker and amplicon sequencing platform, provided error profiles are characterized using positive control sequences. APDP is freely available (http://www.ict.csiro.au/downloads.php) and is distributed with additional scripts for converting APDP outputs into QIIME and mothur formats for downstream diversity analyses.

## Supporting Information

Figure S1 **Workflow of APDP.** An example is shown for the validation of three real sequences from their pyrosequence and PCR errors (indels [blue], polymerase errors [purple], and chimeras [red]), distributed among four samples (A–D).
(DOCX)

Figure S2 **Experimental design used to generate the six 18Smock pyrosequenced amplicon data sets.** Step 1: Mock assemblages (18Smock1-6) created by mixing differing dilutions of 16 plasmid clones of 18S rRNA amplicons (see Table S2 for sequences and dilution group information). Plasmids were classified into one of three dilution groups which determined the concentration in each assemblage relative to clones in Group 1. Two very similar sequences (Clones 6A and 6B) were assigned to Group 3, and each added at one-half the concentration of Group 2 to simulate a heterozygotic individual. Step 2: Five independent 18S rRNA PCR amplifications (35 cycles) were performed for each assemblage, resulting in 15 PCRs. For each assemblage, each amplification was performed at a different time point by the same technician. Step 3: Each PCR was then subjected to four further rounds of PCR amplification with fusion primers to add Titanium sequencing adapters and MID (DNA barcode) sequences. This was performed in triplicate with the same forward barcode (denoted by the PCR number) and one of three reverse barcodes (R01–R03), resulting in 45 total PCRs. Step 4: The 45 PCRs were column-purified, quantified by nanodrop, and pooled into a single tube in equal concentrations. Step 5: The pooled sample was split into two, and each half sequenced independently using Roche 454 GS FLX Titanium chemistry. The first half contained data sets 18Smock1-3 and the second half contained data sets 18Smock4-6.
(DOCX)

Figure S3 **Analysis pipeline used for QIIME analyses including Denoiser implementation.** Specific data set filenames have been replaced by xxxx.
(DOCX)

Figure S4 **Analysis pipeline used for mothur analyses including AmpliconNoise implementation (shhh.flows).** Specific data set filenames have been replaced by xxxx. Parameter values are shown for the 18Smock and 18SEnv analyses, with alternative settings for 16S analyses indicated where relevant.
(DOCX)

Figure S5 **Reproducibility of observed sequences among technical replicates.** Columns and positive error bars indicate the mean and standard deviation of the proportion of unique sequences in each 18Smock data set observed in a given number of technical replicates. Black squares and error bars show the average and standard error of the mean rank abundance for each sequence in the 18Smock-6 Assemblage 3) data set for each number of technical replicates (n = 6).
(DOCX)

Figure S6 **Rank abundance of top 120 sequences from the 18Smock-6 data set.** a. The top 120 sequences based on total read abundance. b.-f. Top 120 sequences from each technical replicate (1–5). Y-axis is log-scaled. Yellow bars indicate reference sequences validated in final analysis, blue bars are error sequences rejected by APDP.
(DOCX)

Table S1 **Roche 454 GS FLX Titanium amplicon pyrosequencing data sets used in this study.** Shown are the number of samples in the dataset, DNA marker amplified, origin of the data set, median expected amplicon length of available reference sequences, total number of raw reads returned, number of assigned reads (based on matches to primers and barcodes), and whether the data set was used to train or test APDP.
(DOCX)

Table S2 **Sequences used to create 18Smock assemblages, including clone names, dilution group assignments for relative concentrations, and plasmid insert sequence.**
(DOCX)

Table S3 **Availability of previously published datasets used in this study.**
(DOCX)

Table S4 **Relative frequencies of 18S rRNA amplicon sequences from known decapod species in both environmental data sets and the number of decapods sequences recovered by APDP.** For each dataset, samples were pooled by forward MID for analysis.
(DOCX)

Table S5 **The observed relative frequencies for each clone sequence in the 18Smock data sets.** The group for each clone is also shown. Maximum and minimum frequencies for each assemblage are shown in bold.
(DOCX)

Table S6 **Classification of reference sequences based on denoised pyrosequences from two single-sample datasets for which APDP performed poorly: (a) 16Sv34; and (b) 16Sv6.** The number of reads in the raw data that match each reference is shown. Results for each method include near-match sequences as correct (“TP+NM”) and are shown before and after application of a read abundance cutoff “(c)”. AN = AmpliconNoise. White = true positive; light gray = near-match; dark gray = false positive (no denoised pyrosequence within 3% sequence similarity); black = false negative (missed).
(DOCX)

Table S7 **Effect of sequence truncation length on the accuracy of denoised pyrosequences and 3% OTUs retained by APDP and AmpliconNoise analyses of six 16Sv13 datasets.** TP = true positive, MC = miscalled, NM = near-match, FP = false positive, FN = false negative.
(DOCX)

Table S8 **Accuracy of OTUs retained by alternative approaches to error removal.** Expected and observed numbers of OTUs assigned to each taxon are shown, as well as the number of OTUs falsely assigned to other decapod taxa not present in the Murray River.
(DOCX)

Table S9 **Comparison of computational processing time in days required for APDP, QIIME and mothur to analyse three pyrosequence datasets.** For APDP, the proportion of the total processing time taken up by the BLAST group assignment step is shown in parentheses. All analyses were performed on the same desktop PC except the 18SEnv1 mothur analysis, which was run on a six-core 80 GB RAM computer.
(DOCX)

Table S10 **Effect of alternative methods used to assign unique sequences to groups on the accuracy of denoised pyrosequences and 3% OTUs retained by APDP.** TP = true positive, MC = miscalled, NM = near-match, FP = false positive, FN = false negative.
(DOCX)
