# Supplementary material for: Improved Inference of Taxonomic Richness from Environmental DNA
Source: PLoS One. 2013 Aug 26;8(8):e71974. doi: 10.1371/journal.pone.0071974 (PMC3753314; doi:10.1371/journal.pone.0071974)

**Figure S1.** Workflow of APDP. An example is shown for the validation of three real sequences from their pyrosequence and PCR errors (indels [blue], polymerase errors [purple], and chimeras [red]), distributed among four samples (A-D).


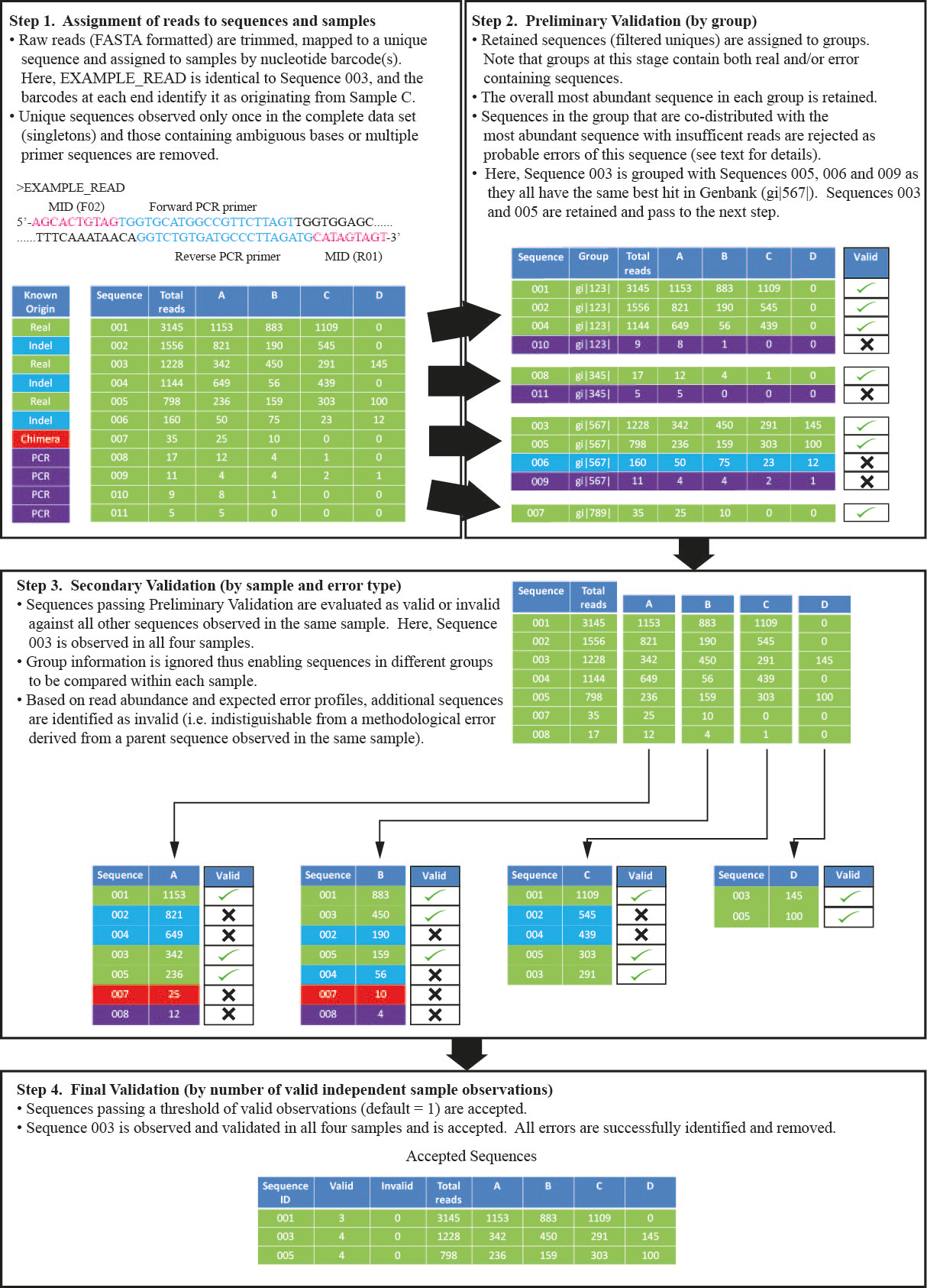

Supplement: Figure S1 — Workflow of APDP. An example is shown for the validation of three real sequences from their pyrosequence and PCR errors (indels [blue], polymerase errors [purple], and chimeras [red]), distributed among four samples (A–D). (DOCX) [file pone.0071974.s001.docx]
