# Supplementary material for: Improved Inference of Taxonomic Richness from Environmental DNA
Source: PLoS One. 2013 Aug 26;8(8):e71974. doi: 10.1371/journal.pone.0071974 (PMC3753314; doi:10.1371/journal.pone.0071974)

**Figure S2.** Experimental design used to generate the six 18Smock pyrosequenced amplicon data sets.

Step 1: Mock assemblages (18Smock1-6) created by mixing differing dilutions of 16 plasmid clones of 18S rRNA amplicons (see Table S2 for sequences and dilution group information). Plasmids were classified into one of three dilution groups which determined the concentration in each assemblage relative to clones in Group 1. Two very similar sequences (Clones 6A and 6B) were assigned to Group 3, and each added at one-half the concentration of Group 2 to simulate a heterozygotic individual.

Step 2: Five independent 18S rRNA PCR amplifications (35 cycles) were performed for each assemblage, resulting in 15 PCRs. For each assemblage, each amplification was performed at a different time point by the same technician.

Step 3: Each PCR was then subjected to four further rounds of PCR amplification with fusion primers to add Titanium sequencing adapters and MID (DNA barcode) sequences. This was performed in triplicate with the same forward barcode (denoted by the PCR number) and one of three reverse barcodes (R01-R03), resulting in 45 total PCRs.

Step 4: The 45 PCRs were column-purified, quantified by nanodrop, and pooled into a single tube in equal concentrations.

Step 5: The pooled sample was split into two, and each half sequenced independently using Roche 454 GS FLX Titanium chemistry. The first half contained data sets 18Smock1-3 and the second half contained data sets 18Smock4-6.


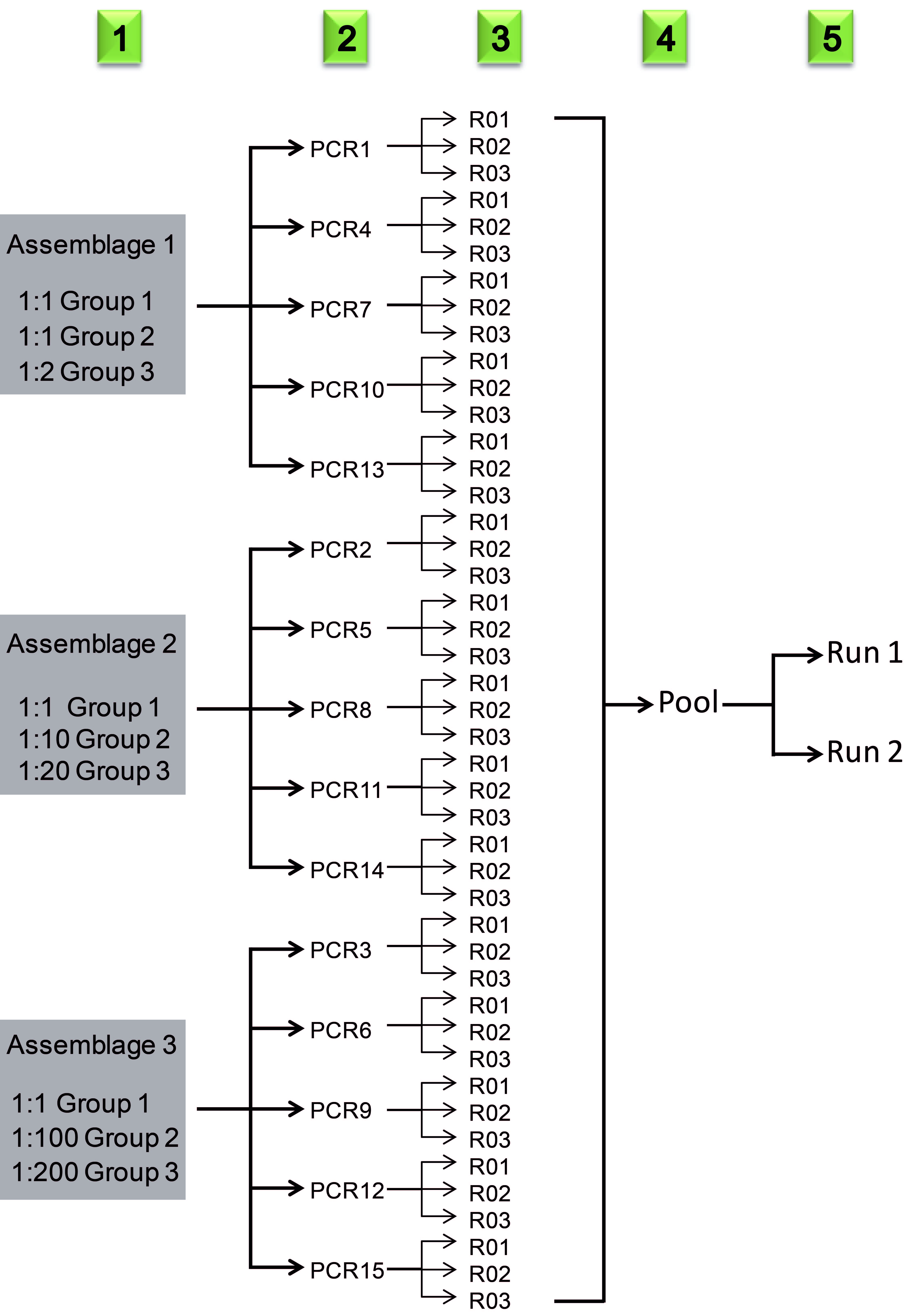

Supplement: Figure S2 — Experimental design used to generate the six 18Smock pyrosequenced amplicon data sets. Step 1: Mock assemblages (18Smock1-6) created by mixing differing dilutions of 16 plasmid clones of 18S rRNA amplicons (see Table S2 for sequences and dilution group information). Plasmids were classified into one of three dilution groups which determined the concentration in each assemblage relative to clones in Group 1. Two very similar sequences (Clones 6A and 6B) were assigned to Group 3, and each added at one-half the concentration of Group 2 to simulate a heterozygotic individual. Step 2: Five independent 18S rRNA PCR amplifications (35 cycles) were performed for each assemblage, resulting in 15 PCRs. For each assemblage, each amplification was performed at a different time point by the same technician. Step 3: Each PCR was then subjected to four further rounds of PCR amplification with fusion primers to add Titanium sequencing adapters and MID (DNA barcode) sequences. This was performed in triplicate with the same forward barcode (denoted by the PCR number) and one of three reverse barcodes (R01–R03), resulting in 45 total PCRs. Step 4: The 45 PCRs were column-purified, quantified by nanodrop, and pooled into a single tube in equal concentrations. Step 5: The pooled sample was split into two, and each half sequenced independently using Roche 454 GS FLX Titanium chemistry. The first half contained data sets 18Smock1-3 and the second half contained data sets 18Smock4-6. (DOCX) [file pone.0071974.s002.docx]
