# Supplementary material for: Improved Inference of Taxonomic Richness from Environmental DNA
Source: PLoS One. 2013 Aug 26;8(8):e71974. doi: 10.1371/journal.pone.0071974 (PMC3753314; doi:10.1371/journal.pone.0071974)
Supplement: Figure S3 — Analysis pipeline used for QIIME analyses including Denoiser implementation. Specific data set filenames have been replaced by xxxx. (DOCX) [file pone.0071974.s003.docx]

**Figure S3.** Analysis pipeline used for QIIME analyses including Denoiser implementation. Specific data set filenames replaced the blanked out text (xxxx).

sffinfo xxxx.sff > xxxx.sff.txt

sffinfo -s xxxx.sff > xxxx.trim.fasta

sffinfo -q xxxx.sff > xxxx.trim.qual

split_libraries.py -o xxxx -f xxxx.fna -q xxxx.qual -m xxxx_mapping.txt -w 50 -l 150 -b 10

denoise_wrapper.py -n 3 -v -i xxxx.sff.txt -f xxxx/seqs.fna -o xxxx/denoised/ -m xxxx_mapping.txt --titanium

inflate_denoiser_output.py -c xxxx/denoised/centroids.fasta -s xxxx/denoised/singletons.fasta -f xxxx/seqs.fna -d xxxx/denoised/denoiser_mapping.txt -o xxxx_denoised_seqs.fna

#custom perl script used to remove remaining reverse primer and MID sequences for 18Smock and 18SEnv data sets.

#pick otus for chimera checking

pick_otus.py -s 0.97 -i xxxx_denoised_seqs.fna -m uclust --optimal

pick_rep_set.py -i uclust_picked_otus/xxxx_denoised_seqs_otus.txt -f xxxx_denoised_seqs.fna -o xxxx_denoised_97id_rep_set.fna

#align representative sequences with pynast and run chimeraslayer

align_seqs.py –i xxxx_rep_set.fna –t core_Silva_aligned.fasta -o pynast_aligned/ -e 100

identify_chimeric_seqs.py -m ChimeraSlayer -i xxxx_rep_set_aligned.fna –a core_Silva_aligned.fasta -o chimeric_seqs.txt

#made OTUs with denoised, chimera-checked pyrosequences and picked representative sequences.

pick_otus.py -s 0.97 -i xxxx_denoised_chimerachecked.fna -m uclust --optimal

pick_rep_set.py –i uclust_picked_otus/xxxx_denoised_chimerachecked_otus.txt –f xxxx_denoised_chimerachecked.fna –o rep_set.fna

#made OTU table

make_otu_table.py -i uclust_picked_otus/xxxx_denoised_chimerachecked_otus.txt -o xxxx_otu_table.txt
