# Supplementary material for: Improved Inference of Taxonomic Richness from Environmental DNA
Source: PLoS One. 2013 Aug 26;8(8):e71974. doi: 10.1371/journal.pone.0071974 (PMC3753314; doi:10.1371/journal.pone.0071974)
Supplement: Figure S4 — Analysis pipeline used for mothur analyses including AmpliconNoise implementation (shhh.flows). Specific data set filenames have been replaced by xxxx. Parameter values are shown for the 18Smock and 18SEnv analyses, with alternative settings for 16S analyses indicated where relevant. (DOCX) [file pone.0071974.s004.docx]

**Figure S4.** Analysis pipeline used for mothur analyses including AmpliconNoise implementation (shhh.flows). Specific data set filenames replaced the blanked out text (xxxx). Parameter values are shown for the 18Smock and 18SEnv analyses, with alternative settings for 16S analyses indicated where relevant.

sffinfo(sff=xxxx.sff, flow=T)

summary.seqs(fasta=xxxx.fasta)

trim.flows(flow=xxxx.flow, oligos=primers.txt, minflows=200, processors=3) #minflows=60-300 for 16S datasets

mpirun -np 3 mothurMPI "#shhh.flo ws(file=xxxx.flow.files)" #run from command line

shhh.flows(file=xxxx.flow.files, processors=6)

trim.seqs(fasta=xxxx.shhh.fasta, name=xxxx.shhh.names, oligos=primers.txt)

unique.seqs(fasta=current, name=current)

summary.seqs(fasta=current, name=current)

align.seqs(fasta=current, reference=silva.eukarya.fasta) #silva.bacteria.fasta for 16S datasets

screen.seqs(fasta=current, name=current, group=current, start=34336, end=37820, minlength=80) #parameters for 16S datasets varied

filter.seqs(fasta=current, vertical=T, trump=.)

unique.seqs(fasta=current, name=current)

pre.cluster(fasta=current, name=current, group=current, diffs=2)#diffs value ~2% of sequence length for 16S datasets

chimera.uchime(fasta=current, name=current, group=current)

remove.seqs(accnos=current, fasta=current, name=current, group=current)

dist.seqs(fasta=current, cutoff=0.20)

cluster(column=xxxx.shhh.trim.unique.good.filter.unique.precluster.pick.dist, name=xxxx.shhh.trim.unique.good.filter.unique.precluster.pick.names, method=average)

#Made OTU table for 3% OTUs

make.shared(list=xxxx.shhh.trim.unique.good.filter.unique.precluster.pick.an.list, group=xxxx.shhh.good.pick.groups, label=0.03)

#Obtained representative sequences.

get.oturep(column=xxxx.shhh.trim.unique.good.filter.unique.precluster.pick.dist, name=xxxx.shhh.trim.unique.good.filter.unique.precluster.pick.names, fasta=xxxx.shhh.trim.unique.good.filter.unique.precluster.pick.fasta, list=xxxx.shhh.trim.unique.good.filter.unique.precluster.pick.an.list, label=0.03, sorted=size)
