# Supplementary material for: Improved Inference of Taxonomic Richness from Environmental DNA
Source: PLoS One. 2013 Aug 26;8(8):e71974. doi: 10.1371/journal.pone.0071974 (PMC3753314; doi:10.1371/journal.pone.0071974)

**Figure S5.** Reproducibility of observed sequences among technical replicates. Columns and positive error bars indicate the mean and standard deviation of the proportion of unique sequences in each 18Smock data set observed in a given number of technical replicates. Black squares and error bars show the average and standard error of the mean rank abundance for each sequence in the 18Smock-6 Assemblage 3) data set for each number of technical replicates (n=6).


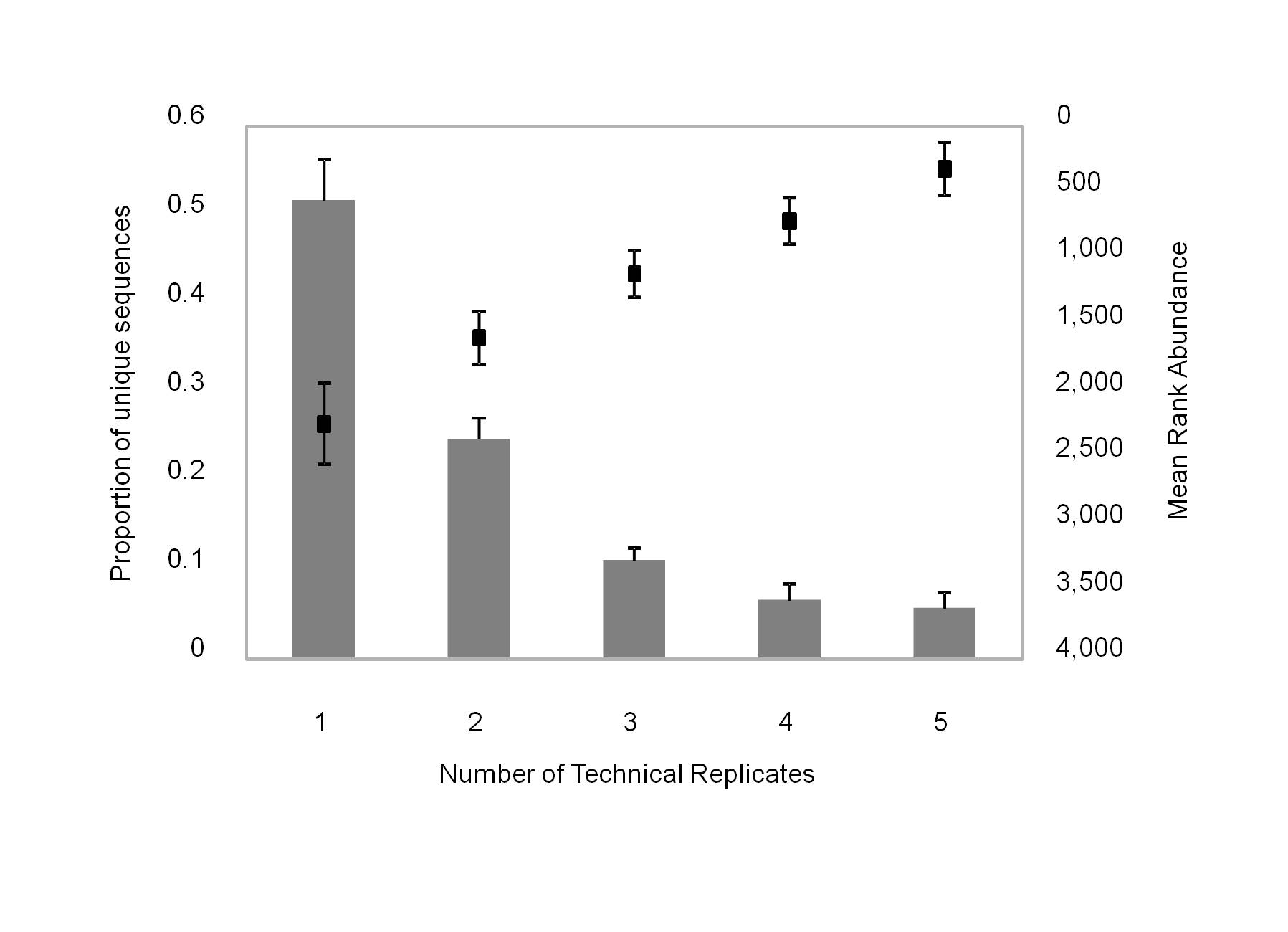

Supplement: Figure S5 — Reproducibility of observed sequences among technical replicates. Columns and positive error bars indicate the mean and standard deviation of the proportion of unique sequences in each 18Smock data set observed in a given number of technical replicates. Black squares and error bars show the average and standard error of the mean rank abundance for each sequence in the 18Smock-6 Assemblage 3) data set for each number of technical replicates (n = 6). (DOCX) [file pone.0071974.s005.docx]
