# Supplementary material for: Improved Inference of Taxonomic Richness from Environmental DNA
Source: PLoS One. 2013 Aug 26;8(8):e71974. doi: 10.1371/journal.pone.0071974 (PMC3753314; doi:10.1371/journal.pone.0071974)

**Figure S6.** Rank abundance of top 120 sequences from the 18Smock-6 data set. **a.** The top 120 sequences based on total read abundance. **b.-f.** Top 120 sequences from each technical replicate (1-5). Y-axis is log-scaled. Yellow bars indicate reference sequences validated in final analysis, blue bars are error sequences rejected by APDP.


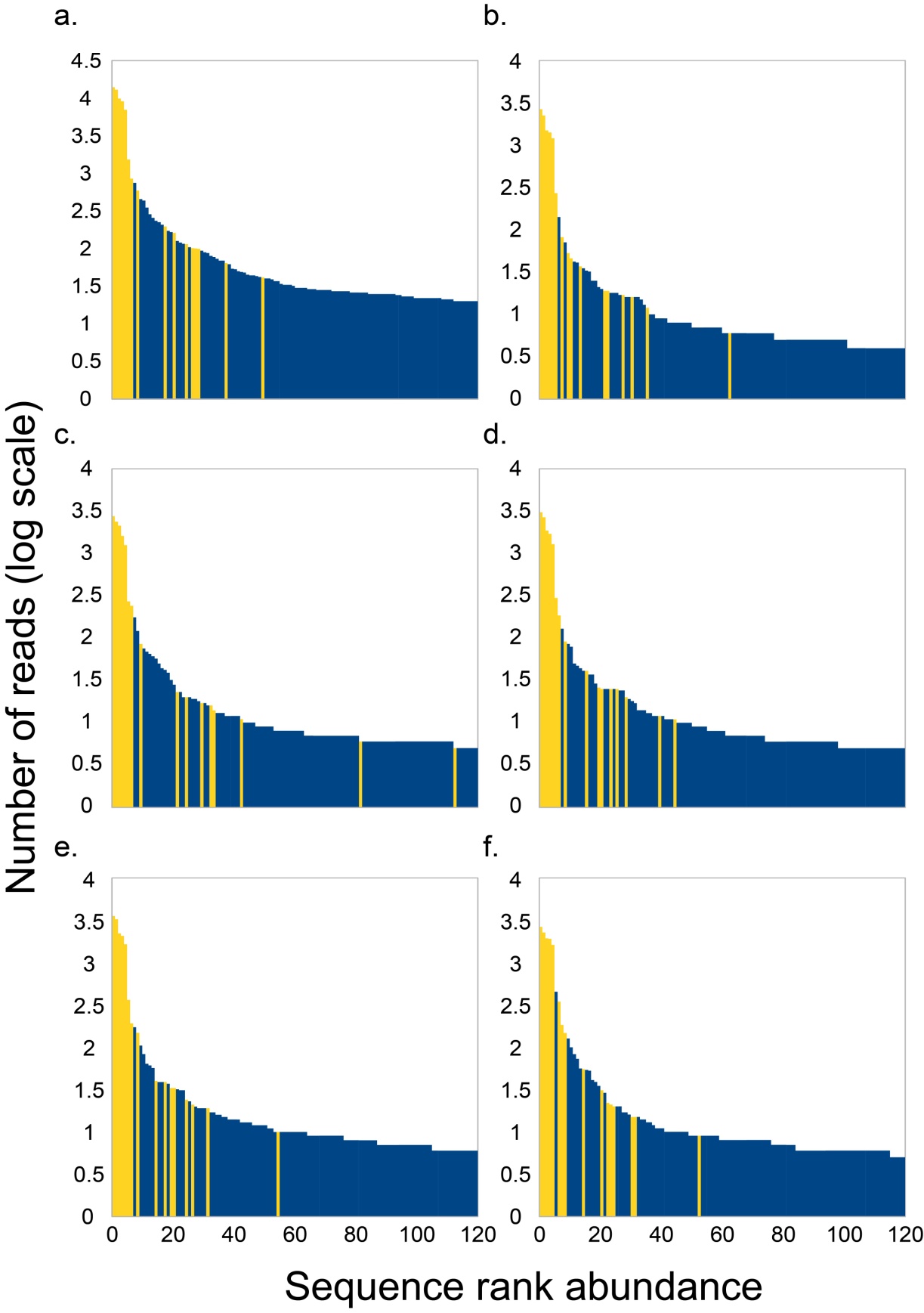

Supplement: Figure S6 — Rank abundance of top 120 sequences from the 18Smock-6 data set. a. The top 120 sequences based on total read abundance. b.-f. Top 120 sequences from each technical replicate (1–5). Y-axis is log-scaled. Yellow bars indicate reference sequences validated in final analysis, blue bars are error sequences rejected by APDP. (DOCX) [file pone.0071974.s006.docx]
