# Supplementary material for: Improved Inference of Taxonomic Richness from Environmental DNA
Source: PLoS One. 2013 Aug 26;8(8):e71974. doi: 10.1371/journal.pone.0071974 (PMC3753314; doi:10.1371/journal.pone.0071974)
Supplement: Table S1 — Roche 454 GS FLX Titanium amplicon pyrosequencing data sets used in this study. Shown are the number of samples in the dataset, DNA marker amplified, origin of the data set, median expected amplicon length of available reference sequences, total number of raw reads returned, number of assigned reads (based on matches to primers and barcodes), and whether the data set was used to train or test APDP. (DOCX) [file pone.0071974.s007.docx]

**Table S1.** Roche 454 GS FLX Titanium amplicon pyrosequencing data sets used in this study. Shown are the number of samples in the dataset, DNA marker amplified, origin of the data set, median expected amplicon length of available reference sequences, total number of raw reads returned, number of assigned reads (based on matches to primers and barcodes), and whether the data set was used to train or test APDP.

| Dataset | Samples | Marker | Origin | Median amplicon length (bp) | Total reads | Accepted reads | Type | Source |
| --- | --- | --- | --- | --- | --- | --- | --- | --- |
| High diversity |  |  |  |  |  |  |  |  |
| 18SEnv1 | 44 | 18S | Environmental | 152 | 574,526 | 361,778 | Test | This study |
| 18SEnv2 | 48 | 18S | Environmental | 152 | 378,898 | 317,243 | Test | This study |
| Low Diversity |  |  |  |  |  |  |  |  |
| 18Smock1-3 | 45 | 18S | 16 clones | 139 | 268,874 | 208,077 | Training | This study |
| 18Smock4-6 | 45 | 18S | 16 clones | 139 | 274,876 | 221,964 | Test | This study |
| 16Sv13 | 12 | 16S | 21 prokaryotes | 491 | 268,874 | 260,161 | Test | [1] |
| 16Sv34 | 1 | 16S | 20 clones | 407 | 75,477 | 11,729 | Test | [2] |
| 16Sv45 | 1 | 16S | 91 clones | 374 | 62,873 | 22,793 | Test | [3] |
| 16Sv6 | 1 | 16S | 20 clones | 60 | 53,654 | 40,205 | Test | [2] |

[1] Jumpstart Consortium Human Microbiome Project Data Generation Working Group (2012) Evaluation of 16S rDNA-Based Community Profiling for Human Microbiome Research. PLoS ONE 7(6): e39315. doi:10.1371/journal.pone.0039315

[2] Lee et al., 2012 (V3V4P)

[3] Quince et al., 2011 (Titanium)
