# Supplementary material for: Improved Inference of Taxonomic Richness from Environmental DNA
Source: PLoS One. 2013 Aug 26;8(8):e71974. doi: 10.1371/journal.pone.0071974 (PMC3753314; doi:10.1371/journal.pone.0071974)
Supplement: Table S2 — Sequences used to create 18Smock assemblages, including clone names, dilution group assignments for relative concentrations, and plasmid insert sequence. (DOCX) [file pone.0071974.s008.docx]

**Table S2.** Sequences used to create 18Smock assemblages, including clone names, dilution group assignments for relative concentrations, and plasmid insert sequence.

| Name | Group | Sequence (5’-3’) |
| --- | --- | --- |
| Clone01 | 1 | TGGTGGAGCGATTTGTCTGGTTAATTCCGATAACGAACGAGACTCTAGCCTGCTAAATAGTACGCCGATCTTTTGTCGGCGCGTACTTCTTAGAGGGACAAGTAGCGGTTAGCTACACGAAATTGAGCAATAACA |
| Clone02 | 1 | GGTGGAGTGATTTGTCTGGTTAATTCCGATAACGAACGAGACTCTGTCCTGCTAACTAGTGTCTGCTTCATTCGTTGAGCAGGTCATTCTTCTTAGAGGGACTGGTAGCTCTTAGCTACACGAGATTGAGCAATAACA |
| Clone03 | 2 | TGGTGGAGTGATTTGTCTGGTTAATTCCGATAACGAACGAGACTCCGTCCTGCTAACTAGTTGGGAGGTCACCCTTTGCCTCCCAAATTCTTCTTAGAGGGACTGACAGCATCTAGCTGTACGAGATGGAGCAATAACA |
| Clone04 | 1 | TGGTGGAGTGATTTGTCTGGTTAATTCCGATAACGAACGAGACCCCGTCCTACTTACTAGACGGAGGTTTCTTTTGACCTCCGAGTCTTCTTAGAGGGACAACTGGCGTTTATCTAGCTACATGAAATGGGGCAATAACA |
| Clone05 | 2 | TGGTGGAGTGATTTGTCTGGTTAATTCCGATAACGAACGAGACTCTGGCCTACTAAATAGACGGCGGATCTTTGTGTCCGTTCTGTCTTCTTAGAGGGATAAGGCAGCTCGAAGCTGCGTGAAAAGGGGCAATAACA |
| Clone06A | 3 | TGGTGGAGCGATTTGTCTGGTTAATTCCGATAACGAACGAGACTCTAGCCTGCTAAATAGTACGCCGATCTTTAGTCGGCGCGTACTTCTTAGAGGGGCAAGTAGCGGATAGCTACACGAAATTGAGCAATAACG |
| Clone06B | 3 | TGGTGGAGCGATTTGTCTGGTTAATTCCGATAACGAACGAGACTCTAGCCTGCTAAATAGTACGCCGATCTTTAGTCGGCGCGTACTTCTTAGAGGGGCAAGTAGCGGATAGCTACACGAAATTGAGCAATAACA |
| Clone08 | 1 | TGGTGGAGCGATTTGTCTGGTTAATTCCGATAACGAACGAGACTCTAGCCTGCTAAATAGGTGTCGGGTTTTTACATCTCCTCGCAAGGGGAGAGTGGAGTGTGTGGTTGTCGGGATCCTGGGCTGTGATGGTCGTTTAGGCTGCGGGGGCAACTTCGTGGTCTGGCCGGCTGTCATAGTTCACCCGGCGTACGCACACTTCGCGTCCGGCATTTTTCTTCTTAGAGGGACAAGCGGCGTCCAGCCGCGTGACAGTGAGCAATAACA |
| Clone09 | 2 | TGGTGGAGTGATTTGTCTGGTTAATTCCGTTAACGAACGAGACCTTAACCTGCTAAATAGTTACGCTAACTCTGGTTGGCGCTTAACTTCTTAGAGGGACTATGGATTTTTAATCCATGGAAGTTTGAGGCAATAACA |
| Clone10 | 1 | TGGTGGGTTGTCTTGTCAGGTTGATTCCGGTAACGAACGAGACCTCAGCCTTTAAATAGTCACTGTCGCTTTTTGCGGCTGGCTTTTGACTTCTTAGAGGGACAGTTGGCGTTTAGTCAACGGAAGTATGAGGCAATAACA |
| Clone11 | 1 | TGGTGGAGTGATTTGTCTGGTTAATTCCGTTAACGAACGAGACCTCAGCCTGCTAAATAATGGTATGAATGATTCGTCATTGATAGCCGTTTCTTAGAGGGACATGGAGTTTTAAGCTCCAGGAAGATTGAGGCAATAACA |
| Clone12 | 2 | TGGTGGAGTGATTTGTCTGGTTAATTCCGTTAACGAACGAGACCCCCGCCTGCTAAATAGACTGGTGAATGATTCTTCATTAACCTGGTCTTCTTAGAGGGACTTTTGGTGACTAACCGAAGGAAGTTGGGGGCAATAACA |
| Clone13 | 1 | TGGTGGAGTGATTTGTCTGGTTTATTCCGATAACGAGCGAGACTCTAGCCTATTAAATAGTCAACGGATAAACGCGTCCGTGTCGACTTCTTAGAGGGACAAGCGGTGTTCAGCCGCATGAAGTTGAGCAATAACA |
| Clone14 | 2 | TGGTGGAGCGATTTGTCTGGTTAATTCCGATAACGAACGAGACTCTAGCCTGCTAAATAGACAGGGAATCGTTAGTTCTCTGTACTTCTTAGAGGGACAAGCGGCATCTAGCTGCACGAGACAGAGCAATAACA |
| Clone15 | 1 | TGGTGGGTTGCCTTGTCAGGTTGATTCCGGTAACGAACGAGACCTCAGCCTGCTAAATAGTCACGGTCGCTCCTTGTGGCCGGCTGCACTTCTTAGAGGGACTATTAGCGTCTAGCTAATGGAAGTATGAGGCAATAACA |
| Clone16 | 2 | TGGTGGGTTGCCTTGTCAGGTTGATTCCGGTAACGAACGAGACCTCAGCCTGCTAAATAGTCACTTTCGCTTTTTGCGGATGGCAGACTTCTTAGAGGGACAAATGACGTTCCAGTCATTGGGAGTTTGAGGCAATAACA |
