# Supplementary material for: Improved Inference of Taxonomic Richness from Environmental DNA
Source: PLoS One. 2013 Aug 26;8(8):e71974. doi: 10.1371/journal.pone.0071974 (PMC3753314; doi:10.1371/journal.pone.0071974)
Supplement: Table S3 — Availability of previously published datasets used in this study. (DOCX) [file pone.0071974.s009.docx]

**Table S3.** Availability of previously published datasets used in this study.

| Dataset | Source | Accessions |
| --- | --- | --- |
| 16Sv13 | NCBI SRA | SRR042563 SRR042565 SRR042567 SRR042572 SRR042574 SRR042576  SRR042587 SRR042589 SRR042591 SRR042593 SRR042595 SRR042597 |
| 16Sv34 | EBI SRA | ERR159950 |
| 16Sv45 | NCBI SRA | SRR068370 |
| 16Sv6 | EBI SRA | ERR159953 |
