# Supplementary material for: Improved Inference of Taxonomic Richness from Environmental DNA
Source: PLoS One. 2013 Aug 26;8(8):e71974. doi: 10.1371/journal.pone.0071974 (PMC3753314; doi:10.1371/journal.pone.0071974)
Supplement: Table S4 — Relative frequencies of 18S rRNA amplicon sequences from known decapod species in both environmental data sets and the number of decapods sequences recovered by APDP. For each dataset, samples were pooled by forward MID for analysis. (DOCX) [file pone.0071974.s010.docx]

**Table S4.** Relative frequencies of 18S rRNA amplicon sequences from known decapod species in both environmental data sets and the number of decapods sequences recovered by APDP. For each dataset, samples were pooled by forward MID for analysis.

| Data Set | *Caridina mccullochi* | *Macrobrachium australiense* | *Paratya australiensis* | Recovered by APDP |
| --- | --- | --- | --- | --- |
| 18SEnv1 | | | | |
| Total | 2.465324% | 0.082924% | 0.001935% | 3 |
| Sample1 | 0.038541% | 0% | 0% | 1 |
| Sample2 | 12.055740% | 0.002068% | 0% | 2 |
| Sample3 | 2.281429% | 0.681466% | 0.015954% | 3 |
| Sample4 | 0.004890% | 0% | 0% | 1 |
| Sample5 | 0.009145% | 0% | 0% | 1 |
| Sample6 | 0.017591% | 0% | 0% | 1 |
| Sample7 | 5.793841% | 0% | 0% | 1 |
| Sample8 | 0.002180% | 0% | 0% | 1 |
| Sample9 | 0.002646% | 0% | 0% | 1 |
| 18SEnv2 | | | | |
| Total | 8.148643% | 0% | 0.014500% | 2 |
| Sample1 | 0% | 0% | 0% | 0 |
| Sample2 | 15.727194% | 0% | 0% | 1 |
| Sample3 | 0.113617% | 0% | 0.014820% | 2 |
| Sample4 | 0.050347% | 0% | 0% | 1 |
| Sample5 | 0% | 0% | 0% | 0 |
| Sample6 | 0% | 0% | 0% | 0 |
| Sample7 | 0.005742% | 0% | 0.001914% | 2 |
| Sample8 | 0.084127% | 0% | 0.084127% | 2 |
| Sample9 | 0% | 0% | 0.071975% | 1 |
| Sample10 | 56.520205% | 0% | 0% | 1 |
| Sample11 | 0.016697% | 0% | 0% | 1 |
| Sample12 | 51.095687% | 0% | 0% | 1 |
