# Supplementary material for: Improved Inference of Taxonomic Richness from Environmental DNA
Source: PLoS One. 2013 Aug 26;8(8):e71974. doi: 10.1371/journal.pone.0071974 (PMC3753314; doi:10.1371/journal.pone.0071974)
Supplement: Table S5 — The observed relative frequencies for each clone sequence in the 18Smock data sets. The group for each clone is also shown. Maximum and minimum frequencies for each assemblage are shown in bold. (DOCX) [file pone.0071974.s011.docx]

**Table S5.** The observed relative frequencies for each clone sequence in the 18Smock data sets. The group for each clone is also shown. Maximum and minimum frequencies for each assemblage are shown in bold.

|  |  | 18Smock1-3 | | | 18Smock4-6 | | |
| --- | --- | --- | --- | --- | --- | --- | --- |
| Clone Name | Group | A1 | A2 | A3 | A4 | A5 | A6 |
| Clone01 | 1 | 4.42% | 9.58% | 11.12% | 4.40% | 9.59% | 11.44% |
| Clone02 | 1 | 3.39% | 8.01% | 9.43% | 3.07% | 7.28% | 8.84% |
| Clone03 | 2 | 6.75% | 1.63% | 0.20% | **8.05%** | 1.95% | 0.25% |
| Clone04 | 1 | 5.78% | 12.48% | 14.42% | 7.07% | **14.21%** | **17.67%** |
| Clone05 | 2 | **8.26%** | 2.05% | 0.24% | 7.54% | 2.10% | 0.21% |
| Clone06A | 3 | 3.69% | 0.76% | 0.10% | 4.11% | 0.83% | 0.08% |
| Clone06B | 3 | 3.37% | 0.77% | 0.07% | 3.35% | 0.83% | 0.13% |
| Clone08 | 1 | 0.78% | 2.33% | 2.05% | 0.77% | 2.18% | 1.92% |
| Clone09 | 2 | 4.39% | 1.15% | 0.20% | 4.46% | 1.10% | 0.14% |
| Clone10 | 1 | 3.54% | 8.24% | 10.35% | 4.11% | 9.58% | 12.49% |
| Clone11 | 1 | **0.26%** | **0.71%** | 0.79% | **0.24%** | **0.62%** | 0.74% |
| Clone12 | 2 | 1.66% | 0.96% | **0.04%** | 2.36% | 1.48% | **0.05%** |
| Clone13 | 1 | 7.03% | **14.67%** | **17.51%** | 7.05% | 13.70% | 16.33% |
| Clone14 | 2 | 2.94% | 0.98% | 0.09% | 2.53% | 0.83% | 0.13% |
| Clone15 | 1 | 0.69% | 0.99% | 1.08% | 0.71% | 0.95% | 1.07% |
| Clone16 | 2 | 5.37% | 1.27% | 0.15% | 6.17% | 1.38% | 0.13% |
| Mean |  | 3.89% | 4.16% | 4.24% | 4.12% | 4.29% | 4.48% |
| SD |  | 2.35% | 4.74% | 6.07% | 2.50% | 4.86% | 6.48% |
