# Supplementary material for: Improved Inference of Taxonomic Richness from Environmental DNA
Source: PLoS One. 2013 Aug 26;8(8):e71974. doi: 10.1371/journal.pone.0071974 (PMC3753314; doi:10.1371/journal.pone.0071974)
Supplement: Table S6 — Classification of reference sequences based on denoised pyrosequences from two single-sample datasets for which APDP performed poorly: (a) 16Sv34; and (b) 16Sv6. The number of reads in the raw data that match each reference is shown. Results for each method include near-match sequences as correct (“TP+NM”) and are shown before and after application of a read abundance cutoff “(c)”. AN = AmpliconNoise. White = true positive; light gray = near-match; dark gray = false positive (no denoised pyrosequence within 3% sequence similarity); black = false negative (missed). (DOCX) [file pone.0071974.s012.docx]

**Table S6.** Classification of reference sequences based on denoised pyrosequences from two single-sample datasets for which APDP performed poorly: (a) 16Sv34; and (b) 16Sv6. The number of reads in the raw data that match each reference is shown. Results for each method include near-match sequences as correct (“TP+NM”) and are shown before and after application of a read abundance cutoff “(c)”. AN = AmpliconNoise. White = true positive; light gray = near-match; dark gray = false positive (no denoised pyrosequence within 3% sequence similarity); black = false negative (missed).

A)

| Reference | Reads | APDP | AN | AN(c) | mothur | mothur(c) | qiime | qiime(c) |
| --- | --- | --- | --- | --- | --- | --- | --- | --- |
| A32 | 889 |  |  |  |  |  |  |  |
| A16 | 333 |  |  |  |  |  |  |  |
| A34 | 248 |  |  |  |  |  |  |  |
| A09 | 165 |  |  |  |  |  |  |  |
| A36 | 143 |  |  |  |  |  |  |  |
| A10 | 15 |  |  |  |  |  |  |  |
| A08 | 9 |  |  |  |  |  |  |  |
| A38 | 262 |  |  |  |  |  |  |  |
| A05 | 128 |  |  |  |  |  |  |  |
| J02 | 47 |  |  |  |  |  |  |  |
| A35 | 21 |  |  |  |  |  |  |  |
| A14 | 0 |  |  |  |  |  |  |  |
| A31 | 0 |  |  |  |  |  |  |  |
| A06 | 1 |  |  |  |  |  |  |  |
| A12 | 1 |  |  |  |  |  |  |  |
| A04 | 0 |  |  |  |  |  |  |  |
| A11 | 0 |  |  |  |  |  |  |  |
| A13 | 0 |  |  |  |  |  |  |  |
| A15 | 0 |  |  |  |  |  |  |  |
| A17 | 0 |  |  |  |  |  |  |  |

B)

| Reference | Reads | APDP | AN | AN(c) | mothur | mothur(c) | qiime | qiime(c) |
| --- | --- | --- | --- | --- | --- | --- | --- | --- |
| A32 | 7686 |  |  |  |  |  |  |  |
| A16 | 4905 |  |  |  |  |  |  |  |
| A34 | 2997 |  |  |  |  |  |  |  |
| A09 | 5059 |  |  |  |  |  |  |  |
| A36 | 2362 |  |  |  |  |  |  |  |
| A10 | 321 |  |  |  |  |  |  |  |
| A08 | 1 |  |  |  |  |  |  |  |
| A38 | 4793 |  |  |  |  |  |  |  |
| A05 | 3835 |  |  |  |  |  |  |  |
| J02 | 342 |  |  |  |  |  |  |  |
| A35 | 1930 |  |  |  |  |  |  |  |
| A14 | 129 |  |  |  |  |  |  |  |
| A31 | 39 |  |  |  |  |  |  |  |
| A06 | 1 |  |  |  |  |  |  |  |
| A12 | 6 |  |  |  |  |  |  |  |
| A04 | 2 |  |  |  |  |  |  |  |
| A11 | 7 |  |  |  |  |  |  |  |
| A13 | 1 |  |  |  |  |  |  |  |
| A15 | 3 |  |  |  |  |  |  |  |
| A17 | 0 |  |  |  |  |  |  |  |
