# Supplementary material for: Improved Inference of Taxonomic Richness from Environmental DNA
Source: PLoS One. 2013 Aug 26;8(8):e71974. doi: 10.1371/journal.pone.0071974 (PMC3753314; doi:10.1371/journal.pone.0071974)
Supplement: Table S7 — Effect of sequence truncation length on the accuracy of denoised pyrosequences and 3% OTUs retained by APDP and AmpliconNoise analyses of six 16Sv13 datasets. TP = true positive, MC = miscalled, NM = near-match, FP = false positive, FN = false negative. (DOCX) [file pone.0071974.s013.docx]

**Table S7.** Effect of sequence truncation length on the accuracy of denoised pyrosequences and 3% OTUs retained by APDP and AmpliconNoise analyses of six 16Sv13 datasets. TP = true positive, MC = miscalled, NM = near-match, FP = false positive, FN = false negative.

| Method | Truncation length (bp) | Expected sequences | Observed sequences | TP | NM | FP | FN | Expected OTUs | Observed OTUs | TP | MC | NM | FP | FN |
| --- | --- | --- | --- | --- | --- | --- | --- | --- | --- | --- | --- | --- | --- | --- |
| APDP | 200 | 24 | 34 | 23 | 0 | 11 | 1 | 18 | 20 | 18 | 0 | 0 | 2 | 0 |
| AmpliconNoise | 200 | 24 | 264 | 21 | 0 | 243 | 3 | 18 | 115 | 17 | 1 | 0 | 97 | 0 |
| cutoff | 200 | 24 | 57 | 21 | 0 | 36 | 3 | 18 | 45 | 17 | 1 | 0 | 29 | 0 |
|  |  |  |  |  |  |  |  |  |  |  |  |  |  |  |
| APDP | 400 | 45 | 47 | 35 | 1 | 11 | 9 | 19 | 27 | 18 | 0 | 1 | 8 | 0 |
| AmpliconNoise | 400 | 45 | 1050 | 21 | 8 | 1021 | 16 | 19 | 377 | 15 | 3 | 1 | 358 | 0 |
| cutoff | 400 | 45 | 118 | 21 | 3 | 94 | 21 | 19 | 129 | 15 | 3 | 1 | 110 | 0 |
