# Supplementary material for: Improved Inference of Taxonomic Richness from Environmental DNA
Source: PLoS One. 2013 Aug 26;8(8):e71974. doi: 10.1371/journal.pone.0071974 (PMC3753314; doi:10.1371/journal.pone.0071974)
Supplement: Table S8 — Accuracy of OTUs retained by alternative approaches to error removal. Expected and observed numbers of OTUs assigned to each taxon are shown, as well as the number of OTUs falsely assigned to other decapod taxa not present in the Murray River. (DOCX) [file pone.0071974.s014.docx]

**Table S8.** Accuracy of OTUs retained by alternative approaches to error removal. Expected and observed numbers of OTUs assigned to each taxon are shown, as well as the number of OTUs falsely assigned to other decapod taxa not present in the Murray River.

| Dataset and Method | *Caridina muccullochi* | *Macrobrachium australiense* | *Paratya australiensis* | Other Decapods | Total Decapod OTUs |
| --- | --- | --- | --- | --- | --- |
| 18SEnv1 |  |  |  |  |  |
| Expected | 1 | 1 | 1 | 0 | 3 |
| APDP | 1 | 1 | 1 | 0 | 3 |
| QIIME | 7 | 1 | 1 | 1 (1 species) | 10 |
| mothur | 125 | 32 | 7 | 20 (5 species) | 184 |
| 18SEnv2 |  |  |  |  |  |
| Expected | 1 | 0 | 1 | 0 | 2 |
| APDP | 1 | 0 | 1 | 0 | 2 |
| QIIME | 1 | 0 | 0 | 0 | 1 |
| mothur | 91 | 0 | 3 | 0 | 94 |
