# Supplementary material for: Improved Inference of Taxonomic Richness from Environmental DNA
Source: PLoS One. 2013 Aug 26;8(8):e71974. doi: 10.1371/journal.pone.0071974 (PMC3753314; doi:10.1371/journal.pone.0071974)
Supplement: Table S9 — Comparison of computational processing time in days required for APDP, QIIME and mothur to analyse three pyrosequence datasets. For APDP, the proportion of the total processing time taken up by the BLAST group assignment step is shown in parentheses. All analyses were performed on the same desktop PC except the 18SEnv1 mothur analysis, which was run on a six-core 80 GB RAM computer. (DOCX) [file pone.0071974.s015.docx]

**Table S9.** Comparison of computational processing time in days required for APDP, QIIME and mothur to analyse three pyrosequence datasets. For APDP, the proportion of the total processing time taken up by the BLAST group assignment step is shown in parentheses. All analyses were performed on the same desktop PC except the 18SEnv1 mothur analysis, which was run on a six-core 80GB RAM computer.

| Dataset | APDP | QIIME | mothur |
| --- | --- | --- | --- |
| 18SEnv1 | 8.52 (57%) | 5.12 | 3.64 |
| 18Smock1 | 0.68 (99%) | 0.08 | 0.12 |
| 16Smock | 0.36 (96%) | 0.10 | 0.25 |
