# Supplementary material for: Improved Inference of Taxonomic Richness from Environmental DNA
Source: PLoS One. 2013 Aug 26;8(8):e71974. doi: 10.1371/journal.pone.0071974 (PMC3753314; doi:10.1371/journal.pone.0071974)
Supplement: Table S10 — Effect of alternative methods used to assign unique sequences to groups on the accuracy of denoised pyrosequences and 3% OTUs retained by APDP. TP = true positive, MC = miscalled, NM = near-match, FP = false positive, FN = false negative. (DOCX) [file pone.0071974.s016.docx]

**Table S10.** Effect of alternative methods used to assign unique sequences to groups on the accuracy of denoised pyrosequences and 3% OTUs retained by APDP. TP = true positive, MC = miscalled, NM = near-match, FP = false positive, FN = false negative.

|  |  | Sequences | | | | OTUs | | | |
| --- | --- | --- | --- | --- | --- | --- | --- | --- | --- |
|  |  | TP only | | TP+NM | | TP only | | TP+NM | |
| Dataset | Denoising algorithm (group assignment method) | FP | FN | FP | FN | FP | FN | FP | FN |
| 16Sv13 | SLP | 6 | 5 | 6 | 5 | 3 | 0 | 3 | 0 |
|  | BLAST | 11 | 1 | 11 | 1 | 2 | 0 | 2 | 0 |
| 16Sv45 | SLP | 10 | 7 | 6 | 3 | 6 | 6 | 3 | 3 |
|  | BLAST | 17 | 4 | 13 | 0 | 4 | 6 | 1 | 3 |
